# Retention of patients in opioid substitution treatment: A systematic review

**DOI:** 10.1371/journal.pone.0232086

**Published:** 2020-05-14

**Authors:** Aisling Máire O’Connor, Gráinne Cousins, Louise Durand, Joe Barry, Fiona Boland

**Affiliations:** 1 School of Pharmacy and Biomolecular Sciences, Royal College of Surgeons in Ireland, Dublin, Ireland; 2 Population Health Medicine, Public Health and Primary Care, Trinity College Dublin, Dublin, Ireland; 3 Data Science Centre, Royal College of Surgeons in Ireland, Dublin, Ireland; Johns Hopkins University Bloomberg School of Public Health, UNITED STATES

## Abstract

**Background:**

Retention in opioid substitution (OST) treatment is associated with substantial reductions in all cause and overdose mortality. This systematic review aims to identify both protective factors supporting retention in OST, and risk factors for treatment dropout.

**Methods:**

A systematic search was performed using MEDLINE, Embase, PsycInfo, CINAHL and Web of Science (January 2001 to October 2019). Randomised controlled trials (RCTs) and observational cohort studies reporting on retention rates and factors associated with retention in OST were included. Factors associated with treatment retention and dropout were explored according to the Maudsley Addiction Profile. A narrative synthesis is provided.

**Results:**

67 studies were included in this review (4 RCTs and 63 observational cohort studies; N = 294,592), all assessing factors associated with retention in OST or treatment dropout. The median retention rate across observational studies was approximately 57% at 12 months, which fell to 38.4% at three years. Studies included were heterogeneous in nature with respect to treatment setting, type of OST, risk factor assessment, ascertainment of outcome and duration of follow-up. While the presence of such methodological heterogeneity makes it difficult to synthesise results, there is limited evidence to support the influence of a number of factors on retention, including age, substance use, OST drug dose, legal issues, and attitudes to OST.

**Conclusions:**

Younger age, substance use particularly cocaine and heroin use, lower doses of methadone, criminal activity/incarceration, and negative attitudes to MMT appear to be associated with reduced retention in OST. A consensus definition of retention is required to allow for comparability across future studies.

## Introduction

Opioid dependence is a serious public health problem, contributing substantially to the global disease burden. The number of people with opioid dependence worldwide increased from 18.2 million in 1990 to 26.8 million in 2016. Furthermore, the years of life lost attributable to opioid dependence was estimated at 3.6 million in 2016 [1]. Leading causes of death among people with opioid dependence include unintentional drug overdose, suicide, HIV and Hepatitis C infection [2]. North America is currently in the midst of an opioid crisis, with escalating opioid overdose deaths initially attributable to prescription opioid use, and more recently to the epidemic of illicit heroin use and illicitly manufactured fentanyl [3, 4]. The opioid crisis represents an urgent challenge to reduce harms associated with opioids. Effective treatments are essential to address the emerging public health threats associated with opioid dependence and opioid overdose.

Opioid substitution treatment (OST), either with methadone or buprenorphine, is the first line treatment for opioid dependence [5, 6], as it has been shown to be safe and effective in suppressing illicit opioid use [7, 8], improving mental and physical well-being [9, 10], and reducing mortality, especially overdose deaths [11]. However, growing evidence suggests that mortality risk remains high during the first 4 weeks of treatment initiation and treatment cessation [11–14]. As a full opioid agonist, methadone can cause hazardous respiratory depression and is associated with an excess risk of death from overdose during the first four weeks of treatment initiation, relative to the remainder of time on treatment [11–13, 15, 16]. Buprenorphine, a partial opioid receptor agonist, is associated with a reduced risk of opioid overdose at treatment initiation when compared to methadone [14, 17]. The mortality risk in the first four weeks following cessation of OST, with either buprenorphine or methadone, is high [13, 14, 16, 18] and could exceed 30 deaths/1000 person years [15]. While careful clinical assessment of opioid tolerance prior to induction onto methadone and continued monitoring during the induction phase may reduce the risk of mortality at treatment initiation, retaining patients in OST, either methadone or buprenorphine, will reduce the risk of exposure to mortality after cessation of OST.

Previous systematic reviews of retention in OST focused on drug dosing strategies [8, 19–21] with or without comparisons of medications (e.g. buprenorphine versus methadone) [8, 19–22]. No systematic review to date has comprehensively investigated factors associated with both retention and cessation rates in OST. One systematic review examined risk factors associated with dropout from addiction treatment, reporting on 122 studies which included an active psychosocial treatment between 1992 and 2013 [23]. The most consistent risk factors for dropout across studies were cognitive deficits, low treatment alliance, personality disorder, and younger age. With the exception of younger age, demographic factors were not identified as consistent risk factors [23]. As this review only included studies with an active psychosocial treatment, these findings may not be representative of OST. Given that OST is first-line treatment for opioid dependence [5, 6], and retention in OST is associated with substantial reductions in the risk for all cause and overdose mortality [15], a comprehensive assessment of retention in OST is warranted. Any such assessment needs to consider studies examining retention and studies examining dropout, as they are in-fact two sides of the same coin; staying in treatment versus dropping out of treatment. A systematic review of retention rates in methadone maintenance treatment (MMT) in China, identified a number of non-treatment related factors (socio-demographics, support system and social function, economic status and psychological status) and treatment-related factors (methadone dose, drug use, methadone use, MMT clinics, MMT participation, awareness of MMT and HIV sero-status) [24]. Other systematic reviews on this topic were limited to specific factors associated with dropout and retention in MMT; specifically gender [25], drug use and sexual behaviours [26]. In relation to the review by Bawor et al. on gender differences in outcomes of MMT, they pooled data from three studies and reported no evidence of gender differences in treatment retention across the three studies [25].

The aim of this study was to conduct a systematic review to identify both protective factors supporting retention in OST, with either methadone or buprenorphine, and risk factors for OST dropout.

## Materials and methods

This systematic review was performed according to Preferred Reporting Items for Systematic reviews and Meta-Analyses (PRISMA) guidelines [27].

### Protocol and registration

A protocol was drafted by the reviewers in preparation for this review, and adhered to throughout; it was not published.

### Eligibility criteria

RCTs and observational cohort studies with a minimum of 6 months follow-up, investigating retention, or dropout, of OST involving first-line pharmacological maintenance treatments for opioid dependence (methadone, buprenorphine, or buprenorphine-naloxone combination) [5] were considered eligible for inclusion. RCTs comparing the effectiveness of different types of OST were excluded. Non-randomized clinical trials, case-control studies, cross sectional surveys, case reports, case series and qualitative research studies were also excluded. Any study that included the use of levo-alpha-acetylmethadol was excluded due to its wide discontinuation of use in the early 2000s. Levo-alpha-acetylmethadol was discontinued as a result of its links to increased risks of ventricular rhythm disorders [28]. Studies that focused on OST for use other than maintenance were excluded, for example, pain management and detoxification. Studies involving patients aged ≥ 18 years from primary care and specialist treatment settings were included. Samples representing subpopulations, such as HIV+ samples, prison populations, pregnant women and institutional settings such as hospitals and residential care were excluded. Treatment outcomes in these settings may be influenced by the environment and as such may be biased in reporting. We also excluded studies which reported on the same outcome (retention/dropout) for the same cohort, due to the risk of bias introduced through multiple testing. No further exclusion criteria were applied to the study samples to ensure that all articles were retrieved without restrictions on demographics of the samples.

The review was restricted to English language articles, published between January 2001 and October 2019. We chose 2001 as the opioid agonist agent, levo-alpha-acetylmethadol (LAAM), was withdrawn from the European market in 2001, and was subsequently withdrawn from all markets [28]. The primary outcomes were retention at a given time (dichotomous variable) or the time a patient was in treatment before dropout (if treatment ceased) or at the end of the study follow-up period (continuous variable). An overview of the exclusion/inclusion criteria are provided in S1 Table.

### Information sources and search

A comprehensive search was performed using MEDLINE, Embase, CINAHL, PsycInfo and Web of Science. The search strategy was developed with the assistance of a librarian. Initially, the main phrases and terms were identified; “opioid substitution treatment”, “treatment retention” and “dropout” and combined to create the search string. The search strategy for each database is available in supplementary material (S2 Table). The search, conducted in November 2017 and updated in October 2019, included title, abstracts and keywords in order to ensure that no relevant studies were omitted.

### Study selection and data extraction

Titles and abstracts of identified studies were reviewed by one reviewer (AMOC) to determine potential eligibility. Full text articles were then independently assessed by two reviewers (AMOC/GC) for those articles considered eligible from title/abstract, or when it was unclear whether a study met the inclusion criteria from title and abstract. Any uncertainty in relation to study eligibility was resolved through discussion with other authors. The following data was independently extracted by two reviewers (AMOC/GC) using a pre-piloted data extraction form (S3 Table): year of publication, country, treatment setting, sample size, study design, demographics (e.g. gender, age), data collection/follow-up period, outcome(s) (retention or dropout), definition of outcome(s), proportion retained in treatment at stated follow-up, reported risk /protective factor(s) investigated, analysis conducted and the associations reported. Adjusted estimates were extracted from the studies where possible. A third author (LD) checked the extracted data.

### Risk of bias in individual studies

Risk of bias was evaluated, by two reviewers (GC/LD), using an adapted form of the Newcastle Ottawa Scale (NOS) (S4 Table) for observational studies, and the Cochrane Risk of Bias tool for RCTs. Within the NOS, a ‘star system’ was developed in which a study is assessed on three broad categories: 1) the selection of study groups; 2) the comparability of groups; and 3) the ascertainment of exposure and outcome. Three sections of the NOS assessment were amended to reflect the use of retention/dropout as the primary outcome. A maximum of 7 stars could be awarded to any one study. A maximum of three stars was awarded in the selection section; we added a star for studies that clearly defined retention/dropout. No star was given if a study failed to provide a clear definition of retention or treatment dropout. A maximum of one star was awarded in the comparability section. This was awarded if the study reported conducting multivariable analysis and stated what factors were included and adjusted for. The outcome section was unaltered, with a maximum of three possible stars. The Cochrane Risk of Bias Tool for RCTs, involved assessing random sequence generation, allocation concealment, selective reporting, blinding of participants, personnel and outcome assessor, and incomplete outcome data.

### Summary measures and synthesis of results

Effect sizes reported in the included studies were reported in a variety of ways (i.e. hazard ratio, odds ratio, risk ratio) and as much information as possible was retrieved from studies. Where a study reported on multiple follow-ups, data on factors explored at all time points (>6 months) were extracted. A narrative synthesis was conducted due to the heterogeneity of the studies included. The Economic and Social Research Council guidance on narrative synthesis was referred to when planning the synthesis [29]. Specifically, a preliminary synthesis was developed by tabulating all studies to give an overview of study characteristics. Studies were then organised using categories from the Maudsley Addiction Profile. The categories were as follows: demographics, substance use, treatment factors, health risk behaviours, health symptoms and social functioning. Variables suitable for inclusion in these categories are presented in Table 1. An ‘other’ category was included to capture additional variables investigated in included studies. The Maudsley Addiction Profile captures six of the eight outcome domains recommended by Wiessing et al. [30]. Within the categories outlined above, where possible, relationships across studies were explored and similarities and differences highlighted.

**Table 1:** Categories of the maudsley addiction profile used to guide this review and examples of variables included within each category.

Category	Variables
Demographics	Age
	Gender
	Location
	Race
Substance use	Poly-drug use
	Cannabis use
	Benzodiazepine use
	Cocaine use
	Heroin use
	Alcohol use
	Amphetamines/ecstasy
	Drug use patterns
Treatment Factors	Medication type
	Dosage
	Previous treatments
	Year of treatment intake
	Treatment setting
	Treatment facilities
Health Risk Behaviour	Sexual behaviour
	Injecting
	Sharing needles
Health Symptoms	Non-fatal overdose
	Mental health
	Physical health
	HIV+/-
	Tuberculosis (TB) +/-
Social Functioning	Residence, work and training, relationships, illegal behaviours
	Marital Status
	Employment status
	Income
	Education
	Living status
	Social Support
	Legal issues

## Results

### Study selection

**Fig 1:**
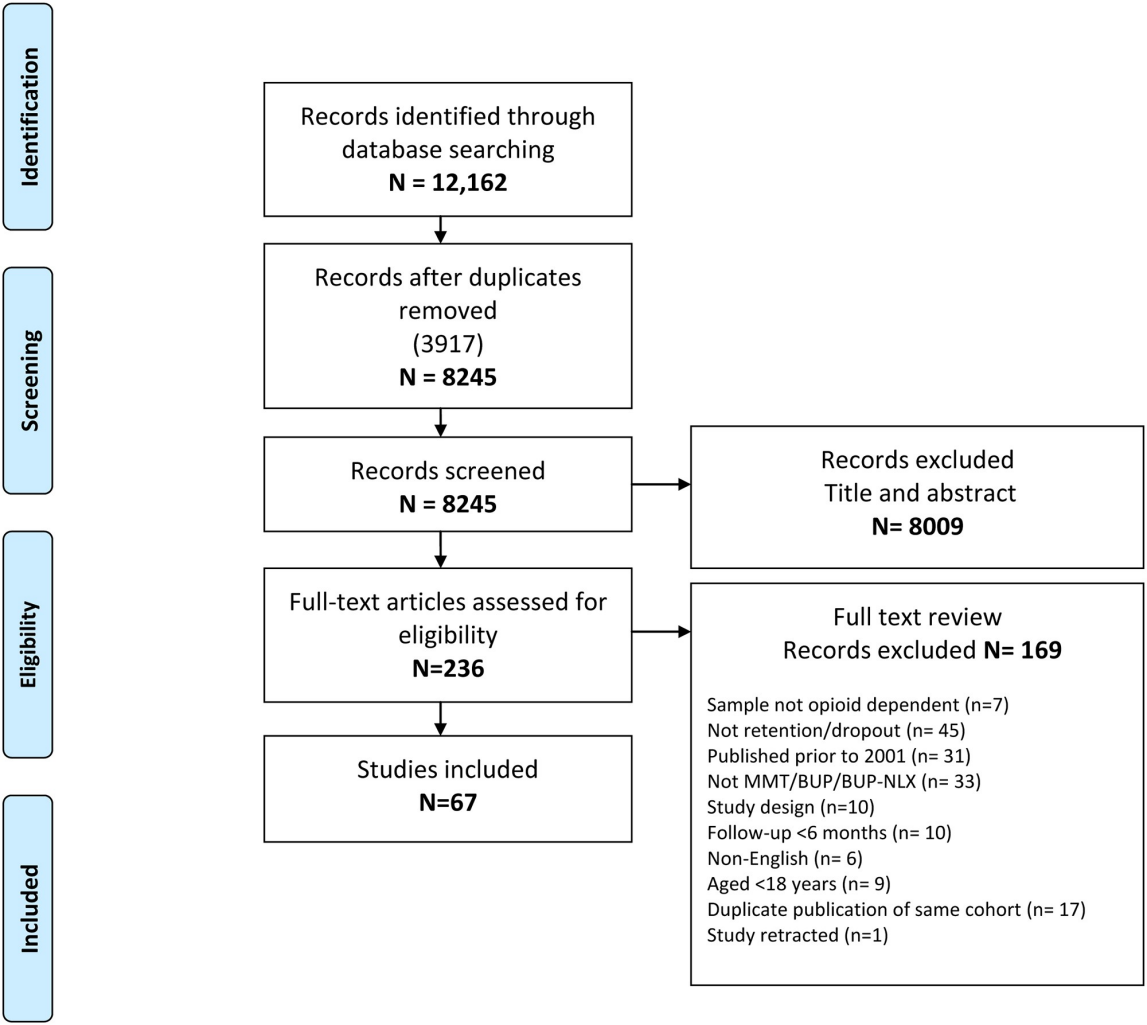
PRISMA flowchart.

Of the 12,162 citations identified from this search strategy, 236 full text articles were assessed for eligibility, with 67 studies meeting the inclusion criteria (Fig 1) [31–97].

### Study characteristics

The main characteristics of included studies are summarised below and presented in full detail in (S5 Table).

#### Country

Studies included in this review were conducted in 21 countries. The majority of studies were conducted in the United States of America (n = 25) [33, 34, 37, 39, 40, 47, 50, 54, 56, 58, 61, 62, 64, 68–72, 75, 79, 82, 84, 86, 88, 93], China (n = 14) [31, 36, 45, 57, 65–67, 80, 90, 92, 94–97], Canada (n = 9) [42, 46, 52, 53, 74, 77, 78, 87, 89] and Europe (n = 9) [32, 38, 41, 43, 48, 51, 55, 59, 73]. The remaining studies were spread across Asia (n = 5) [60, 81, 83, 85, 91], Israel (n = 3) [35, 75, 76], India (n = 1) [49], Australia (n = 1) [44] and Africa (n = 1) [63].

#### Study design

Four of the included studies were randomised controlled trials [31–34]. The majority of the observational studies involved a retrospective cohort study design (n = 37) [37, 39, 42–44, 46, 49–54, 58, 59, 63, 64, 68–70, 72–74, 76, 79–82, 84–87, 89–91, 93, 94, 97], with prospective cohort studies accounting for a further 23 studies [35, 36, 38, 40, 41, 45, 47, 48, 55, 57, 60–62, 65–67, 75, 77, 78, 83, 87, 88, 97]. The remaining three studies involved an ambidirectional cohort study [95] and analyses of data collected as part of an RCT [56, 71].

#### Study setting and OST

There was a large range of treatment settings, with OST clinics accounting for the largest proportion of treatment settings at 53.7% (n = 36) [31, 33–39, 42, 45, 46, 48–51, 53, 55, 57, 60, 62, 64, 66, 67, 75–81, 83, 92, 94–97]. Other treatment settings included office based or primary care OST (n = 9) [32, 40, 47, 58, 82, 84, 87, 88, 93], hospital setting (n = 5) [61, 63, 65, 85, 91], outpatient treatment programmes (n = 4) [54, 56, 71, 72] and mixed treatment settings (n = 3) [41, 44, 73]. The remaining studies reported on national or regional registries, including health insurance databases (n = 10) [43, 52, 59, 69, 74, 86, 89, 90] or Veteran Health Administration records in the US [68, 70]. Two-thirds of the included studies focused on methadone maintenance treatment (MMT) (n = 45) [31–39, 42, 45, 46, 48, 50, 54, 55, 57, 59–67, 73–81, 83, 85, 89–92, 94–97], with 21% focusing on Buprenorphine (n = 14) [40, 47, 49, 56, 58, 68–72, 82, 84, 88, 93]. The remaining studies included mixed OST (patients in study received either MMT or Buprenorphine) (n = 8) [41, 43, 44, 51–53, 86, 87].

#### Participants

There was a combined sample size of 294,592 participants (969 in RCTs and 293,623 in observational studies). Sample sizes ranged from 37 (31) to 107,740 (90). One study reported on the number of treatment episodes (n = 9,555) from a population-based treatment registry, rather than the number of participants [89]. Most studies provided a gender breakdown for their sample, with a higher proportion of men in all but two studies [37, 50]. The mean age of participants ranged from 22.4 years [49] to 47 years [40]

#### Primary outcome

All four RCTs reported on retention [31–34]. While the majority of the observational studies reported on retention rates at various time points (n = 50), the primary outcome for most studies was treatment dropout (n = 39), followed by retention in treatment (n = 24). The most frequently used measure of dropout was time to discontinuation of OST (n = 32), with varying thresholds applied to identify date of dropout. Almost half the studies reporting on time to discontinuation did not specify a threshold for identifying dropout (n = 13) [37, 40, 42, 43, 45, 56, 62, 65, 68, 69, 85, 91, 94]; seven studies indicated that a patient was identified as having dropped out of OST if they missed more than 30 consecutive days of treatment [53, 57, 60, 67, 74, 80, 92]; six studies applied a threshold of seven days [44, 46, 49, 78, 96, 97], and the remaining studies applied a threshold of five days [83], ten days [51], 14 days [58, 66, 95], 21 days [63]and two months [59]. The other six studies reporting on dropout used a binary measure, which identified patients as having dropped out of treatment after a specific period of follow-up [38, 48, 55, 61, 79, 88]. The majority of studies measuring retention in treatment (n = 18) used a binary measure [39, 41, 47, 50, 52, 54, 70–73, 75, 77, 81, 82, 84, 86, 89, 93], with six studies using a continuous measure of days in treatment [35, 36, 64, 76, 87, 90]. The duration of follow-up varied across studies, ranging from 6 months to 24 years (S5 Table).

### Risk of bias within studies

The assessment of risk of bias for the included observational studies is detailed in (S6 Table). The greatest risk of bias was selection bias, with 29 studies reporting on a select group (e.g. a single treatment site/clinic, veterans only) which may undermine the external validity [35–38, 40, 42, 46, 47, 49, 61–65, 68–72, 75–79, 85, 87, 88, 91, 93]. In relation to internal validity, the risk of misclassifying the outcome was considered to be high or unclear for 17 studies [37, 40, 43, 55, 56, 59, 62, 65, 68, 69, 76, 81, 84, 85, 88, 91, 94]. As previously noted, many studies did not specify when dropout was ascertained, that is how many consecutive days without OST were tolerated before a patient was considered to have left treatment. The potential risk of misclassification bias for exposure variables was identified as relatively low, with only four studies identified as having a high risk of misclassifying exposure [35, 64, 83, 85]. Similarly, five studies did not clearly identify what covariates were adjusted for in their multivariable analyses [35, 48, 55, 56, 88]. All studies were identified as having an appropriate duration of follow-up, with adequate follow-up of participants (complete follow-up or <20% attrition and description of loss provided). All four RCTs were considered to have a low risk of selection bias, as participants were randomised to groups [31–34]. Furthermore, intervention allocations could not have been foreseen in advance of or during enrolment for two trials [31, 34], this was unclear for Jaffray et al. and Marsch et al. [32, 33]. The risk of selective outcome reporting was considered to be low for each study. However, the risk of performance bias was high in three of the trials due to a lack of blinding [31, 33, 34]. While Jaffray et al. did not blind participants, they used a cluster trial design which controls for contamination between intervention and control participants [32]. The risk of detection bias was considered to be unclear for three trials [31–33], with Schwartz et al. identified as high risk as the outcome assessor was unblinded at follow-up [34]. Each of the trials was considered to have a low risk of attrition bias.

### Synthesis of results

#### Rates of retention and dropout

As previously stated most studies reported on retention rates, even when the primary outcome analysed was dropout. Median retention rates for various time points and by type of OST are reported in Table 2. As expected, median retention rates decreased as duration of follow-up increased with overall median retention rates declining from 58% at 6 months to 38.4% at 3 years. Median retention rates across type of OST can be compared at 6 and 12 months follow-up. While retention was lower at 12-months for all types of OST, median retention rates were highest in studies involving MMT at both time points. Median retention rates were lowest in mixed OST studies. Consistent with these findings, reported dropout rates increased over time. For example, Sullivan et al. reported a dropout rate of 53% from MMT at 6 months, which increased to 66% at 12 months and 77% at 24 months [90]. With a longer duration of follow-up Zhang et al. reported a dropout rate of 46.3% from MMT at 12 months, which increased to 58.8% at 24 months and 87.6% at seven years [95].

**Table 2:** Median retention rates across included studies.

	Retention 6 months % (range)	Retention 12 months % (range)	Retention 2 years % (range)	Retention 3 years % (range)
**MMT**	67.0% (46.8%–86.0%)	60.7% (20.3%–94.0%)	49.8% (29.5%–76.0%)	54.0% (20.0%–82.0%)
	[n = 9] (63, 65, 66, 74, 75, 78, 79, 81, 83)	[n = 24] (35–37, 46, 48, 54, 55, 59–63, 65, 66, 73–75, 77, 79, 81, 85, 91, 92, 94)	[n = 7] (42, 48, 63, 74, 89, 92, 94)	[n = 6] (37, 46, 59, 91, 92, 94)
**BUP**	56.8% (19.1%–64.0%)	45.4% (11.7%–61.6%)	-	-
	[n = 5] (49, 58, 70, 82, 88)	[n = 6] (49, 68, 69, 72, 84, 93)		
**Mixed OST**	54.0% (52.6%–75.8%)	40.4% (33.0%–65.8%)	-	-
	[n = 5] (44, 47, 51, 86, 87)	[n = 7] (41, 44, 51–53, 86, 87)		
**Overall**	58.0% (19.1%–86.0%)	57.0% (11.7%–94.0%)	49.8% (29.5%–76.0%)	38.4% (13.7%–82.0%)
	[N = 19] (44, 47, 49, 51, 58, 63, 65, 66, 70, 74, 75, 78, 79, 81–83, 86–88)	[N = 37] (35–37, 41, 44, 46, 48, 49, 51–55, 59–63, 65, 66, 68, 69, 72–75, 77, 79, 81, 84–87, 91–94)	[N = 9] (42, 48, 51, 63, 72, 74, 89, 92, 94)	[N = 8] (37, 46, 59, 68, 69, 91, 92, 94)

Median retention rates for buprenorphine and mixed OST are not reported at 2 and 3 years follow-up due to small study numbers (buprenorphine at 2-years (n = 1); buprenorphine at 3 years (n = 2); mixed OST at 2 years (n = 1); mixed OST at 3 years (n = 0)

#### Factors associated with retention or dropout

As noted in S5, the four included RCT’s examined different interventions, including behavioural, drug and HIV risk reduction counselling [31], motivational interviewing for community pharmacists delivering OST [32], a web-based behavioural intervention [33] and different levels of counselling in MMT [34]. None of the four RCTs observed significant effects on patient retention. Results from the individual observational studies assessing the longitudinal association between risk or protective factors and retention are synthesised according to the Maudsley Addiction Profile (Table 3). The number of studies assessing individual factors are presented alongside the number of studies identifying a positive association or increased retention (which refers to a significant increase in retention or a reduction dropout) or a negative association or reduced retention (which refers to a significant decrease in retention or increase in dropout). The results are presented according to the type of OST studied, MMT, Buprenorphine or mixed OST. The full details for each individual study can be seen in S7 Table.

**Table 3:** Synthesis of results by risk factor; reporting total number of studies and number of studies reporting significant effects (direction of effects included).

	MMT			BUP			Mixed OST		
Risk Factor	No. of studies	Ret+/drop-	Ret-/drop+	No. of studies	Ret+/drop-	Ret-/drop+	No. of studies	Ret+/drop-	Ret-/drop+
**Demographics**									
Age (older)	31 (37–39, 42, 50, 54, 59–63, 65, 66, 73–78, 80, 81, 83, 89, 91, 92, 94–97)	18 (37, 39, 50, 54, 59, 63, 74–76, 80, 81, 83, 89, 92, 94, 95, 97)	0	12 (40, 56, 58, 69–72, 82, 84, 86, 88, 93)	8 (56, 58, 69, 70, 72, 82, 86, 93)	0	6 (41, 43, 44, 51, 53, 87)	4 (43, 44, 53, 87)	0
Gender (male)	25 (37–39, 42, 50, 54, 59–63, 65, 66, 73, 74, 77, 78, 81, 85, 90, 92, 95–97)	1 (66)	8 (39, 50, 63, 73, 90, 92, 97)	9 (40, 56, 58, 71, 72, 86, 88, 93, 82)	1 (82)	3 (58, 72, 93)	6 (41, 43, 44, 51, 53, 87)	0	2 (44, 53)
Race (Black/African American)	7 (39, 50, 54, 61, 62, 64)	0	3 (39, 50, 54)	4 (40, 68, 72, 93)	0	2 (68, 93)	0	-	-
Race (Hispanic)	3 (50, 61)	0	0	3 (40, 72, 93)	0	1 (93)	0	-	-
Area deprivation (most deprived)	1 (37)	0	1 (37)	0	-	-	0	-	-
**Substance Use**									
Cannabis	1 (79)	0	0	1 (88)	0	0	1 (87)	1 (87)	0
Benzodiazepine	6 (42, 67, 75, 76, 79)	0	2 (75, 76)	5 (49, 72, 84, 88, 93)	0	0	1 (53)	0	1 (53)
Cocaine	10 (38, 39, 42, 50, 61, 62, 64, 78, 79)	0	7 (39, 42, 50, 64, 78, 79)	6 (40, 56, 58, 71, 88, 93)	0	3 (56, 58, 71)	1(87)	0	0
Heroin/Opiates	8 (54, 65, 75, 76, 79, 80, 83)	0	5 (54, 75, 76, 79, 80)	4 (40, 70, 93)	0	2 (70, 88)	0	0	0
Alcohol	5 (50, 61, 62, 67)	0	0	3 (72, 88, 93)	0	0	1 (87)	1 (87)	0
Amphetamine	8 (39, 50, 65, 67, 75, 79)	0	4 (39, 50, 67, 75)	0	0	0	0	0	0
**Treatment Factors**									
OST Dosage (higher)	26 (36–38, 45, 48, 59, 60, 62, 63, 65, 66, 73–77, 80, 83, 85, 90–92, 94, 95, 97)	23 (36, 37, 45, 48, 62, 63, 65, 66, 73–77, 80, 83, 85, 90–92, 94, 95, 97)	0	3 (49, 56, 88)	1 (49)	1 (56)	1 (51)	1 (51)	0
Treatment setting (specialist addiction clinic/prescriber)	1 (73)	0	1 (73)	1 (86)	0	1 (86)	1 (44)	0	1 (44)
Counselling	1 (62)	0	0	4 (40, 56, 58, 88)	1 (58)	0	0	0	0
Take-home OST doses	3 (59, 83, 85)	2 (83, 85)	0	0	0	0	0	0	0
Previous OST treatments	9 (50, 54, 59, 74, 83, 89, 94)	3 (59, 74, 97)	2 (89, 94)	3 (47, 71, 93)	1 (47)	0	0	0	0
**Health Risk Behaviour**									
Injection drug use	7 (46, 50, 61, 64, 73, 90)	0	1 (46)	2 (49, 56)	1 (49)	0	1(87)	0	1 (87)
Sharing needles	6 (78, 80, 92, 94, 96, 97)	2 (92, 97)	2 (80, 94)	0	0	0	0	0	0
Risky sexual behaviour	2 (63, 78)	0	0	0	0	0	0	0	0
**Health Symptoms**									
Mental health	11 (38, 42, 50, 54, 62, 65, 76, 77, 91, 96)	0	1 (76)	5 (58, 69, 72, 86, 93)	3 (58, 72, 93)	1 (69)	0	0	0
Poor physical health/ increasing comorbidities	7 (39, 54, 59, 62, 74, 91, 96)	2 (62, 74)	2 (39, 96)	2 (68, 86)	0	1 (68)	1 (41)	0	0
HIV +	5 (60, 65, 66, 90, 91)	1 (60)	0	1 (88)	0	0	2 (51, 87)	0	0
HCV+	6 (36, 64, 66, 75, 90, 91)	2 (36, 75)	1 (66)	2 (58, 93)	1 (58)	1 (93)	1 (51)	0	0
Tuberculosis +	1 (65)	0	0	0	0	0	1 (51)	0	0
**Social Functioning**									
Marital status (married/long term partner)	10 (50, 60, 61, 65–67, 90, 95, 97)	4 (60, 67, 90, 97)	0	0	0	0	0	0	0
Employment status (employed/source of income)	14 (50, 54, 60, 62, 65, 66, 74, 78, 85, 90–92, 97)	2 (85, 92)	1 (97)	2 (88, 93)	2 (88, 93)	0	0	0	0
Education (higher)	11 (38, 60, 61, 64–66, 74, 80, 90, 91, 95)	3 (80, 90, 95)	1 (74)	0	0	0	1 (87)	0	0
Homeless	4 (50, 59, 78)	0	1 (59)	0	0	0	1 (87)	0	0
Criminal activity/arrests	14 (39, 46, 50, 54, 62, 65, 66, 77, 78, 83, 90, 91, 94)	0	9 (39, 46, 50, 62, 66, 77, 90, 94)	0	0	0	3 (43, 44, 87)	0	2 (43, 44)
Family support (high)	4 (39, 65, 83, 94)	3 (39, 65, 94)	0	1 (49)	0	0	0	0	0
Contact with other drug users	3 (45, 94, 97)	0	2 (94, 97)	2 (49, 88)	0	1 (49)	0	0	0
**Other**									
Greater distance/time taken to reach OST clinic	8 (37, 50, 60, 65, 66, 90, 97)	0	3 (66, 90, 97)	1 (82)	0	1 (82)	0	0	0
Attitudes to OST (positive)	6 (61, 62, 65, 83, 94, 97)	5 (61, 62, 83, 94, 97)	0	0	0	0	0	0	0

Results relate to observational studies only. Ret+/drop- refers to positive effects such that the factor was associated with increased retention or reduced dropout; Ret-/drop+ refers to negative effects such that the factor associated with reduced retention or increased dropout. MMT (cohorts reporting on methadone maintenance treatment); BUP (cohorts reporting on buprenorphine or buprenorphine-naloxone combination); Mixed OST). *Pele et al. 2008 reported on two cohorts separately (Tel Aviv and Los Angeles) and Deck et al. 2005 reported on two cohorts separately (Oregon and Washington); each cohort is considered as a study in the analysis presented in this table.

*Demographics*. Age was the most frequently studied risk factor; of the 31 MMT studies examining age, 18 studies found increasing age to be associated with increased retention [37, 39, 50, 54, 59, 63, 74–76, 80, 81, 83, 89, 92, 94, 95, 97]. Similar patterns were observed for studies of buprenorphine, with eight of 12 included studies reporting increased retention with age [56, 58, 69, 70, 72, 82, 86, 93], and mixed OST with four of the six included studies reporting consistent effects for age [43, 44, 53, 87]. While gender was widely studied (n = 40), more than half the studies reported a non-significant association between gender and retention. However, where an association was observed, most studies (12/14) identified men as having significantly lower retention [39, 44, 50, 53, 58, 63, 72, 73, 90, 92, 93, 97]. Two studies reported opposite effects, one in relation to MMT [66] and the other Buprenorphine [82]. Race and ethnicity was investigated across 16 studies [37, 39, 40, 50, 54, 61, 62, 64, 68, 72, 87, 88, 90, 91, 93], with most studies reporting on Black or African American (n = 11) [39, 40, 50, 54, 61, 62, 64, 68, 72, 93], White (n = 12) [37, 39, 40, 50, 54, 61, 62, 72, 87, 88, 93], Hispanic (n = 6) ([40, 50, 61, 72, 93] and Native American (n = 3) [39, 50] groups. Of the seven MMT studies reporting on Black or African Americans, six involved a comparison with white participants [39, 50, 54, 61, 62], with one study failing to report the comparison group [64]. Of these seven studies, three reported reduced retention among African American service users relative to White [39, 50, 54]. Similar results were observed in relation to buprenorphine, with two of the four studies reporting reduced retention among African Americans relative to White [93] or other races combined (White/Hispanic/Other) [68]. None of the MMT cohorts reported effects for Hispanic race [50, 61], with only one [93] of the three Buprenorphine cohorts [40, 72, 93] reporting effects such that Hispanic race was associated with reduced retention relative to White. Three MMT cohorts examined Native American relative to White, with no evidence of an effect on retention [39, 50]. Other assessments of race involved a comparison of Han v’s Non-Han in a Chinese cohort, and Malay v’s Non-Malay in a Malaysian cohort, with both studies reporting non-significant effects [90, 91].

Only one study examined area deprivation, identifying a significant association between increasing deprivation and reduced retention in MMT [37].

*Substance use*. Thirty six studies examined the effects of substance use on retention, however, measures of substance use varied greatly across studies ranging from type of drug(s) used, and patterns and frequency of drug use with different recall periods (e.g. lifetime use, last 6 months or last 30 days). As outlined in Table 3 cocaine was most frequently assessed (n = 17) [38–40, 42, 50, 56, 58, 61, 62, 64, 71, 78, 79, 87, 88, 93], followed by benzodiazepines (n = 12) [42, 49, 53, 67, 72, 75, 76, 79, 84, 88, 93], heroin/opiates (n = 12) [40, 54, 65, 70, 75, 76, 79, 80, 83, 88, 93] and amphetamines (n = 8) [39, 50, 65, 67, 75, 79]. Of the ten methadone cohorts reporting on cocaine, seven found cocaine use to be associated with reduced retention [39, 42, 50, 64, 78, 79]. Similarly, three of the six buprenorphine cohorts reported significant effects, with cocaine associated with reduced retention [56, 58, 71]. A smaller number of studies reported significant effects for benzodiazepines; two [75, 76] of the six MMT cohorts reported illicit benzodiazepine use was associated with reduced retention. Similarly, baseline benzodiazepine use was associated with reduced retention in a mixed OST sample [53]. Benzodiazepines were not found to be associated with retention in the buprenorphine cohorts [49, 72, 84, 88, 93]. Amphetamine use was examined in eight methadone cohorts, with half the cohorts reporting a non-significant association (n = 4) [50, 65, 75, 79]; the remaining four cohorts identified reduced retention with amphetamine use [39, 50, 67, 75]. Twelve cohorts reported on heroin/opiate use, eight MMT [54, 65, 75, 76, 79, 80, 83] and four buprenorphine [40, 70, 88, 93]. Five of the MMT cohorts [54, 75, 76, 79, 80] and two of the buprenorphine cohorts [70, 88] reported a significant association, such that heroin/opiate use was associated with reduced retention. Methadone and buprenorphine cohorts reporting on alcohol consumption, all reported no association between alcohol and retention [50, 61, 62, 67, 72, 87, 88, 93]. In contrast, one cohort involving mixed OST, found alcohol consumption to be associated with increased retention at 6 months [87]. Similarly, this cohort was the only study to identify a positive association between cannabis and retention. However, this study was identified as reporting on a select group with self-reported retention.

*Treatment factors*. Dosage was the most frequently studied treatment factor, studied across 26 methadone cohorts [36–38, 45, 48, 59, 60, 62, 63, 65, 66, 73–77, 80, 83, 85, 90–92, 94, 95, 97], 3 buprenorphine cohorts [49, 56, 88] and one mixed OST cohort [51]. However, there was wide variability in measurement of dosage, ranging from average daily dose in categories [45, 60, 74] to dose at treatment initiation [63], or dose after 30 days [66] or at three months [65]. Twenty-three of the 26 methadone cohorts, reported that higher methadone doses were associated with increased retention [36, 37, 45, 48, 62, 63, 65, 66, 73–77, 80, 83, 85, 90–92, 94, 95, 97]. Only three of the buprenorphine cohorts reported on dose, with mixed results [49, 56, 88]; one study found that increase in buprenorphine dose was associated with increased retention [49], with negative effects observed in another cohort [56]. A mixed OST cohort also reported improved retention among those receiving high and medium doses of OST relative to low doses [51].

Three studies reported on the potential effects of treatment setting or treatment provider on retention in MMT [73], buprenorphine [86], and mixed OST [44]. Each study observed a reduction in retention among those attending specialist addiction clinics or prescribers. Mullen et al. found that relative to those attending MMT in primary care, attendance at a specialist treatment centre was associated with reduced retention [73]. Shcherbakova et al.’s buprenorphine cohort also found that being in the care of an addiction specialist was associated with reduced retention [86]. A large Australian cohort of mixed OST found that the effects of treatment setting may be time dependent. They observed that those attending correctional facilities, or community pharmacy, in the first 9 months of treatment, had improved retention relative to those attending an OST clinic. However, after the first 9 months of treatment, those attending correctional facilities had reduced retention relative to those attending an OST clinic, while attending community pharmacies remained protective [44]. Few studies examined counselling, one MMT cohort [62] and four buprenorphine cohorts [40, 56, 58, 88], with only one study reporting counselling as independently associated with retention [58]. Access to take home doses of methadone was also observed to be associated with improved retention in two [83, 85] of the three MMT cohorts examining this factor [59, 83, 85]. A number of studies also considered prior OST experience (n = 12), with mixed results; of the 9 MMT cohorts, four reported non-significant effects [50, 54, 83], with three cohorts suggesting improved retention with prior OST [59, 74, 97] and two cohorts suggesting reduced retention [89, 94]. However, measures of prior treatment varied from prior treatment in the past two years [50], number of prior drug treatments [54, 74, 89], first treatment episode (y/n) [59] and re-enrolled in treatment (y/n) [94, 97]. Only one of the three buprenorphine cohorts reported that those with prior buprenorphine experience has better treatment retention relative to buprenorphine-naïve participants [47].

*Health risk behaviours*. Eighteen studies investigated the association of health risk taking behaviours on retention, 15 involved MMT cohorts [46, 50, 61, 63, 64, 73, 78, 80, 90–92, 94, 96, 97], two buprenorphine [49, 56] and one mixed OST [87]. As shown in Table 3, the most commonly assessed risk behaviour was injection drug use (seven MMT cohorts [46, 50, 61, 64, 73, 90], two buprenorphine [49, 56] and one mixed OST [87]), followed by use of unclean needles or needle sharing (five MMT cohorts [26, 78, 80, 92, 97]), and risky sexual behaviours (three MMT cohorts [63, 78, 91]). Studies reported various time frames of engaging in risk taking behaviours, for example daily injecting [87] or currently injecting [46], injecting in the past 30 days [49, 50, 61], in the six months prior to OST [90] or lifetime injecting (ever inject) [73]. There was no evidence of a relationship between injection drug use and retention in six of the seven MMT cohorts [50, 61, 64, 73, 90]. In contrast, a Canadian cohort of patients in a MMT programme for dependence on opioid analgesics found that current injection drug use was associated with dropout [46]. Socias et al. also found daily heroin injecting to be associated with reduced retention in mixed OST at six months [87]. In contrast, a small buprenorphine cohort in India found past month injection use to be associated with reduced dropout at two years [49]. Mixed results were also observed in relation to sharing needles in MMT; two studies found no association [78, 96], with two studies reporting protective effects such that needle sharing experience was associated with reduced dropout [92, 97]. In contrast, two additional studies identified needle sharing as risk factors for increased treatment dropout [80, 94]. Two MMT cohorts examined risky sexual behaviour, one study assessed condom use [63], while the other assessed working in the sex trade [78]. Both studies reported a non-significant effect.

*Health symptoms*. Mental health status was the most frequently assessed health factor, examined in 11 MMT cohorts [38, 42, 50, 54, 62, 65, 76, 77, 91, 96], and five buprenorphine cohorts [58, 69, 72, 86, 93]. The factors studied varied from psychiatric diagnosis [38, 69, 72, 76, 86, 93], to presence of symptoms or severity of psychiatric symptoms [50, 54, 62, 65, 77, 91, 96], psychiatric treatment history [42] and prescribed psychiatric medications [58]. Only one MMT study found significant effects, such that those with a DSM-IV Axis II diagnosis had reduced retention [76]. Mixed results emerged in relation to buprenorphine, with four of the five buprenorphine cohorts reporting significant effects; three studies found mental health factors to be protective [58, 72, 93], with one study reporting the opposite [69]. Haddad et al. found that patients prescribed psychiatric medications had reduced dropout at 12 months [58], similarly Weinstein et al. found that patients with any psychiatric diagnosis had greater retention at two years [93]. Montalvo’s US cohort also found that patients with depressive disorders and other mood disorders had increased retention at two years [72]. In contrast, Manhapra’s nationwide US study of insured individuals found that patients with any psychiatric diagnosis had increased dropout at three years, however any psychotherapy was associated with reduced dropout during the same time period [69].

Ten studies reported on participants’ physical health status, seven involved MMT cohorts [39, 54, 59, 62, 74, 91, 96], two buprenorphine [68, 86] and one mixed OST [41]. Mixed results were observed across the seven MMT cohorts; three studies reported no significant effects [54, 59, 91], two studies reported that patients with poorer health or greater comorbidities were found to have improved retention [62, 74], with two other studies reporting the opposite effect of reduced retention with poorer self-reported health [39, 96]. Similarly, one buprenorphine cohort found a higher Charlson index (greater number of comorbidities) was independently associated with reduced retention [68]. Variation observed across studies may be an artefact of measurement, as patients’ physical health status was measured differently across studies. Some studies reported on comorbidity scores based on patients’ drug dispensing records [68, 74, 86], while others used physical health scores such as the Addiction Severity Index (medical composite score) [54, 62] or the Short-Form 36 Health Survey [96]. Of the eight studies that investigated HIV status, only one MMT cohort reported an independent association between being HIV + and reduced dropout [60], with all other studies reporting no evidence of an association [51, 65, 66, 87, 88, 90, 91]. Mixed results were observed in relation to Hepatitis C status. Of the six MMT cohorts studying Hepatitis C, two reported that being HCV positive was associated with increased retention [36, 75] with one other study reporting reduced retention [66]. Similarly, of the two buprenorphine cohorts examining HCV status, one study reported positive effects (increased retention) [58] and the other opposing effects [93].

*Social functioning*. Marital or relationship status was explored in ten studies, all involving MMT cohorts [50, 60, 61, 65–67, 90, 95, 97]. Six studies reported no evidence of an association with retention [50, 61, 65, 66, 95, 96]. Three studies reported that being married, cohabiting or being in a long-term relationship was associated with increased retention [60, 90, 97]. A Chinese study also found that being divorced, relative to being single, was associated with lower retention [67].

Employment was explored in fourteen MMT cohorts [50, 54, 60, 62, 65, 66, 74, 78, 85, 90–92, 97], and two buprenorphine [88, 93]. Employment was generally assessed in terms of being employed v’s unemployed [54, 60, 62, 65, 66, 74, 85, 88, 90–93, 97], with two studies reporting on whether participants had a stable source of income [50, 78]. Five of the 16 studies found an association between employment and retention. A Malaysian study of MMT found that full-time employment was associated with reduced dropout compared to unemployment [85]; similar effects were observed in a Chinese MMT cohort [92], with another large Chinese MMT cohort reporting opposing effects as employment was associated with increased dropout [97]. The two buprenorphine cohorts reported consistent findings with employment associated with improved retention [88, 93]. Various levels of education were investigated across 11 MMT cohorts [38, 60, 61, 64–66, 74, 80, 90, 91, 95] and one mixed OST [87]. Three MMT cohorts found that higher levels of education were associated with greater retention [80, 90, 95], with one MMT cohort observing higher levels of education to be associated with increased dropout [74]. However, it is important to note that the latter MMT cohort used ecological data (% of neighbourhood population at various levels of education) for education, not participants’ actual level of education [74].

Four MMT cohorts [50, 59, 78] and one mixed OST [87] examined the effects of homelessness, with only one study observing a significant effect such that no fixed abode or living in an institution was associated with increased dropout [59]. A number of studies would also suggest that living with family is protective; three MMT cohorts found that living with family relative to living with friends or alone was associated with increased retention [90, 92, 97]. Two further studies identified that having children in the home was associated with increased retention [39, 75], an additional study did not provide evidence of this effect [88].

Current and previous legal issues were investigated in 17 studies (14 MMT cohorts [39, 46, 50, 54, 62, 65, 66, 77, 78, 83, 90, 91, 94] and three mixed OST [43, 44, 87]; with eleven studies consistently showing reduced retention associated with criminal activity and arrests/incarceration [39, 43, 44, 46, 50, 62, 66, 77, 90, 94], the remaining studies reported non-significant effects [54, 65, 78, 83, 87, 91]. For example, history of arrests (lifetime) [94], arrested in the past two years [50], incarcerated during study period [44, 66] and increasing number of arrests or criminal charges [43, 46, 62, 77] were associated with reduced retention in treatment. Cox et al. also reported a significant reduction in retention with increasing days of serious conflict with others (excluding family) in the past month [46].

Other social functioning factors considered across studies include family support and contact with other drug users. Four MMT cohorts [39, 65, 83, 94] and one buprenorphine [49] examined the role of family support, with three of the four MMT cohorts reporting increased retention with high family support [39, 65, 94]. Consistent with these findings, Cao et al. also reported that patients who had relatives receiving MMT were more likely to remain in treatment (MMT) [45]. In contrast, contact with other drug users was associated with reduced retention in two MMT cohorts [94, 97] and one buprenorphine cohort [49]. In addition, a study of MMT in Indonesia found that perceived peer support increased the likelihood of dropout [83].

*Other variables investigated in included studies*. A number of studies reported on other variables, covering themes such as distance or time taken to reach OST clinic [37, 50, 60, 65, 66, 82, 90, 97] and attitudes towards OST [61, 62, 65, 83, 94, 97]. Of the nine cohorts reporting on distance or time taken to reach OST clinic, four studies (three MMT cohorts and one buprenorphine) reported significant effects, all suggesting that greater distances or travel time required to reach treatment was associated with reduced retention [66, 82, 90, 97]. Consistent with these findings, Friedmann et al.’s study of 22 MMT clinics in the US also reported that provision of transportation assistance was associated with increased retention [54]. Attitudes to OST were assessed in six MMT cohorts, with positive attitudes to MMT associated with increased retention in five of the six cohorts [61, 62, 83, 94, 97].

## Discussion

### Statement of principal findings

Our systematic review identified 63 observational cohort studies examining factors associated with retention or dropout from OST, and four RCTs assessing the effectiveness of different interventions in improving retention in OST. Retention rates across the observational studies varied widely, with retention rates at 12 months varying from 11.7% (49) to 85.6% (35) across 37 studies. While the National Institute of Drug Abuse (NIDA) recommends a minimum of one year in OST for best outcomes [98], the median retention rate across studies was approximately 57% at 12 months, which fell to 38.4% at three years. Furthermore, differences were observed by type of OST, with methadone cohorts reporting a higher median retention rate at 12 months compared to buprenorphine cohorts and mixed OST cohorts. This finding is consistent with previous studies, suggesting that buprenorphine is associated with shorter duration of treatment relative to methadone [14, 99].

Studies included in this review were heterogeneous in nature with respect to treatment setting, type of OST, risk factor assessment, ascertainment of outcome and duration of follow-up. While the presence of such methodological heterogeneity makes it difficult to synthesise results, there is limited evidence to support the influence of a number of factors on retention, including age, substance use, OST drug dose, legal issues and attitudes to OST. The majority of studies reported significant effects for age, such that older age was associated with increased retention in MMT, buprenorphine and mixed OST. Substance use, particularly cocaine and heroin, were found to have a negative impact on retention in MMT and buprenorphine. Similarly, half the studies examining amphetamine use in MMT, reported reduced retention in treatment. Treatment related factors were most commonly assessed in methadone cohorts. Despite wide variability in assessment of methadone dose, higher doses were consistently observed to be protective. Mixed results were observed in buprenorphine cohorts. Furthermore, two of the three MMT studies investigating take home doses, found that increased take home doses were associated with increased retention. This may be a marker of stability, and is consistent with the finding that increased family support and lower contact with other drug users was associated with increased retention in the majority of MMT studies assessing these factors. In contrast, and reflecting a more chaotic lifestyle, the majority of methadone and mixed OST cohorts investigating legal issues found criminal activity and arrests/incarceration to be associated with reduced retention. Finally, positive attitudes to MMT were associated with increased retention in MMT.

### Strengths and limitations

Our review is the first to synthesise the totality of evidence in relation to factors associated with retention in OST. We used robust and explicit methods to identify, select, appraise and synthesise the study findings. However, the findings of this review need to be considered in the context of the study limitations. Firstly, our study focused on adults aged ≥ 18 years, limiting generalisability of findings to younger cohorts. Secondly, studies had to have a minimum of six months follow-up to be included in this review, which excluded studies of early dropout. Growing evidence suggests that mortality risk is highest in the first four weeks following dropout [13, 16, 18, 100], therefore understanding factors associated with early dropout is important, and could allow for the risk stratification of patients requiring more intensive engagement at the treatment initiation and stabilisation stages. In addition, other risk factors not identified here, such as stress could be important risk factors for early dropout [101]. Thirdly, we restricted our search to the English language, which may have resulted in us missing important studies published in other languages. Finally, we did not carry out a search of the grey literature which may have introduced a potential publication bias.

### Clinical implications and areas for future research

OST, with methadone or buprenorphine, has been shown to be safe and effective in suppressing illicit opioid use, improving physical and mental health, reducing mortality and transmission of HIV and hepatitis C virus, and drug-related crime [2, 20, 102–105]. These protective features of OST are unlikely to be sustained when a person drops out of treatment, particularly if they relapse. Therefore, identifying risk factors for treatment dropout is essential to inform future interventions targeted at retaining patients in treatment. While we identified a large number of studies addressing this question, the overall value of the evidence was diminished due to the lack of comparability across studies arising from variability in the definition of retention or dropout [30]. There does not appear to be an accepted threshold for defining treatment dropout, studies apply various rules ranging from 30 consecutive days without a methadone or buprenorphine prescription, to 21 days, 14 days, 10 days, seven days, five days and up to two months. Furthermore, given the complexity of OST and the fact that patients often cycle in and out of treatment [106, 107], the influence of different risk factors may vary over time, yet few studies consider risk factors as time varying covariates. An international consensus project, using a Delphi methodology could be organised to reach consensus regarding the most clinically appropriate definition of retention. The Delphi methodology allows a consensus opinion to be reached among a panel of experts through an interactive process of questionnaires [108]. A pooled analysis of individual level data from multiple cohorts, particularly those using similar methods to ascertain outcome such as prescription refill data, could also be undertaken to inform an internationally agreed definition. This would allow studies to be replicated and promote scientific progress on this question [30]. Furthermore, as noted by Brorson et al. [23] future studies should provide detail on the treatment process, particularly in relation to involuntary dropout. It is often unclear whether a patient’s dropout was voluntary or involuntary, and while the outcome may be the same the risk factors are likely to be very different. Input from patients and treatment providers may also be beneficial in assessing what are the most pressing issues in retaining patients in treatment.

## Conclusion

Almost half the people in OST are not retained in treatment at 12 months, and this rate reduces further with time. Younger age, substance use, lower doses of methadone, criminal activity/incarceration, and negative attitudes to MMT appear to be associated with reduced retention. A consensus definition of retention is required to allow for comparability across future studies.

## Supporting information

S1 Table Inclusion and exclusion criteria. (DOCX)

S2 Table Search strategy. (DOCX)

S3 Table Data extraction template. (DOCX)

S4 Table Newcastle-Ottawa Scale (NOS) template (adapted). (DOCX)

S5 Table Characteristics of included studies. (DOCX)

S6 Table Results of the critical appraisal of included observational studies using the Newcastle Ottawa Scale (n = 63). (DOCX)

S7 Table Summary of results of included studies; potential risk and protective factors explored. (DOCX)

S1 Checklist PRISMA 2009 checklist. (DOC)
