# Supplementary material for: Retention of patients in opioid substitution treatment: A systematic review
Source: PLoS One. 2020 May 14;15(5):e0232086. doi: 10.1371/journal.pone.0232086 (PMC7224511; doi:10.1371/journal.pone.0232086)
Supplement: S1 Table — (DOCX) [file pone.0232086.s001.docx]

## S1 Table. Inclusion and exclusion criteria

|  | **Inclusion** | **Exclusion** |
| --- | --- | --- |
| **Population** | Adults aged ≥18 years in receipt of opioid substitution treatment for opioid dependence | Pregnant women, HIV patients only |
| **Intervention and OST** | Opioid substitution treatment, with methadone, buprenorphine, or  Buprenorphine-naloxone combination | Opioid substitution therapy for pain management or detox or levo-alpha-acetylmethadol |
| **Setting** | Primary Care, Specialist Treatment | Inpatients, prison, residential care settings |
| **Study Type** | Cohort studies, RCTs | Non-randomized clinical trials, case-control studies, cross sectional surveys, case reports, case series and qualitative research studies, commentary, editorials and responses; duplicate publication of the same cohort |
| **Study Outcome** | Retention or dropout |  |
| **Follow-up period** | at least six months follow-up | less than six months follow-up |
| **Protective or risk factors** | Any factor explored in relation to retention or dropout |  |
