# Supplementary material for: Retention of patients in opioid substitution treatment: A systematic review
Source: PLoS One. 2020 May 14;15(5):e0232086. doi: 10.1371/journal.pone.0232086 (PMC7224511; doi:10.1371/journal.pone.0232086)
Supplement: S2 Table — (DOCX) [file pone.0232086.s002.docx]

## S2 Table. Search strategy

**MEDLINE**

| S1 | "Opioid-Related Disorders"[Mesh] OR "Opiate Substitution Treatment"[Mesh] |
| --- | --- |
| S2 | (Opioid* OR Opiate* OR methadone OR buprenorphine) |
| S3 | "Buprenorphine"[Mesh] OR "Buprenorphine, Naloxone Drug Combination"[Mesh] |
| S4 | "Methadone"[Mesh] |
| S5 | (((("Opioid-Related Disorders"[Mesh] OR "Opiate Substitution Treatment"[Mesh])) OR ((Opioid* OR Opiate* OR methadone OR buprenorphine))) OR ("Buprenorphine"[Mesh] OR "Buprenorphine, Naloxone Drug Combination"[Mesh])) OR "Methadone"[Mesh] (S1 OR S2 OR S3 OR S4) |
| S6 | "Patient Compliance"[Mesh] |
| S7 | complian* OR adherence OR retention OR persisten* |
| S8 | ("Patient Compliance"[Mesh]) OR (complian* OR adherence OR retention OR persisten*) (S6 OR S7) |
| S9 | (((((( "Opioid-Related Disorders"[Mesh] OR "Opiate Substitution Treatment"[Mesh])) OR ((Opioid* OR Opiate* OR methadone OR buprenorphine))) OR ("Buprenorphine"[Mesh] OR "Buprenorphine, Naloxone Drug Combination"[Mesh])) OR "Methadone"[Mesh])) AND (("Patient Compliance"[Mesh]) OR (complian* OR adherence OR retention OR persisten*)) (S8 AND S5) |
|  | Filters: Species – Humans |

**PsycInfo**

| S1 | opioid* OR opiate* OR methadone OR buprenorphine |
| --- | --- |
| S2 | DE" naloxone" OR DE "Buprenorphine" |
| S3 | DE "methadone" OR DE "methadone maintenance" |
| S4 | DE "opiates" OR DE "substance use disorder" OR DE "maintenance therapy" |
| S5 | S1 OR S2 OR S3 OR S4 |
| S6 | complian* OR adherence OR retention OR persisten* |
| S7 | S6 OR S7 |
| S8 | S5 AND S8 |
| S9 | Search Options : Population Group - Human |

**Web of Science**

|  | Query |
| --- | --- |
| 1 | opiate addiction |
| 2 | "opiate addiction" |
| 3 | opioid addiction |
| 4 | "opioid addiction" |
| 5 | opiate substitution |
| 6 | "opiate substitution" |
| 7 | opioid substitution |
| 8 | "opioid substitution" |
| 9 | buprenorphine |
| 10 | opioid maintenance |
| 11 | opioid replacement |
| 12 | methadone |
| 13 | S1 OR S2 OR S3 OR S4 OR S5 OR S6 OR S7 OR S8 OR S9 OR S10 OR S11 OR S12 |
| 14 | Patient adher* |
| 15 | patient complia* |
| 16 | "treatment refusal" |
| 17 | patient retention |
| 18 | treatment dropout |
| 19 | "treatment cessation" |
| 20 | medication adherence |
| 21 | "patient dropout" |
| 22 | S14 OR S15 OR S16 OR S17 OR S18 OR S19 OR S20 OR S21 |
| 23 | S13 AND S22 ^*no option to screen for human only studies^ |

**Embase**

| S1 | ‘opiate addiction'/exp OR 'opiate substitution treatment'/exp |
| --- | --- |
| S2 | ‘buprenorphine' |
| S3 | ‘methadone' |
| S4 | S1 OR S2 OR S3 |
| S5 | 'patient compliance'/exp OR 'patient compliance' |
| S6 | patient retention |
| S7 | ‘patient retention’/exp |
| S8 | ‘treatment refusal'/exp |
| S9 | ‘patient dropout’/exp' or 'patient dropout' |
| S10 | S5 OR S6 OR S7 OR S8 OR S9 |
| S11 | S4 AND S10 |
| S12 | Study type: Human |

**CINHAL**

| S1 | "Opioid-Related Disorders"[Mesh] OR "Opiate Substitution Treatment"[Mesh] |
| --- | --- |
| S2 | Search (Opioid* OR Opiate* OR methadone OR buprenorphine) |
| S3 | Search "Buprenorphine"[Mesh] OR "Buprenorphine, Naloxone Drug Combination"[Mesh] |
| S4 | Search "Methadone"[Mesh] |
| S5 | (((("Opioid-Related Disorders"[Mesh] OR "Opiate Substitution Treatment"[Mesh])) OR ((Opioid* OR Opiate* OR methadone OR buprenorphine))) OR ("Buprenorphine"[Mesh] OR "Buprenorphine, Naloxone Drug Combination"[Mesh])) OR "Methadone"[Mesh] (S1 OR S2 OR S3 OR S4) |
| S6 | Search "Patient Compliance"[Mesh] |
| S7 | Search complian* OR adherence OR retention OR persisten* |
| S8 | Search ("Patient Compliance"[Mesh]) OR (complian* OR adherence OR retention OR persisten*) (S6 OR S7) |
| S9 | Search (((((( "Opioid-Related Disorders"[Mesh] OR "Opiate Substitution Treatment"[Mesh])) OR ((Opioid* OR Opiate* OR methadone OR buprenorphine))) OR ("Buprenorphine"[Mesh] OR "Buprenorphine, Naloxone Drug Combination"[Mesh])) OR "Methadone"[Mesh])) AND (("Patient Compliance"[Mesh]) OR (complian* OR adherence OR retention OR persisten*)) (S8 AND S5) |
|  | Search Options: Human |
