# Supplementary material for: Retention of patients in opioid substitution treatment: A systematic review
Source: PLoS One. 2020 May 14;15(5):e0232086. doi: 10.1371/journal.pone.0232086 (PMC7224511; doi:10.1371/journal.pone.0232086)
Supplement: S3 Table — (DOCX) [file pone.0232086.s003.docx]

## S3 Table. Data extraction template

Reference (authors, year, title, journal): ___________

Setting (country and treatment setting): _____________

OST Type: ____________________

Study Design (RCT/cohort study): _____________

Participants

N: ___________

Sex: ___________

Mean age (±SD): ___________

Age range: ___________

Study Outcome definition(s): ____________________

Duration of follow-up (data collection periods) : ________________

Outcome(s) of interest (retention/dropout): ________________

Time points: ________________

Proportion(s) of sample retained in treatment: ________________

Proportion(s) of sample ceasing treatment (dropout): ________________

Analysis conducted: ________________

Reported risk/protective factors explored and associations reported: ________________
