# Supplementary material for: Retention of patients in opioid substitution treatment: A systematic review
Source: PLoS One. 2020 May 14;15(5):e0232086. doi: 10.1371/journal.pone.0232086 (PMC7224511; doi:10.1371/journal.pone.0232086)
Supplement: S4 Table — (DOCX) [file pone.0232086.s004.docx]

## S4 Table. Newcastle-Ottawa Scale (NOS) template (adapted)

**Please note that within each section a maximum of one star can be given for each numbered item. Hence, a maximum of 3 stars can be awarded for the selection and outcome sections and a maximum of 1 star for the comparability section.**

**Selection**

1) Representativeness of the exposed cohort (Include a small description of the sample)

a) Truly representative of the average OST user in the community (random sample from dataset/medical records)

b) Somewhat representative of the average OST user in the community (non-random sample from dataset e.g. inclusion/exclusion criteria in place)

c) Selected group of users e.g. OST users from 1 specific treatment centre, HIV only, veterans only

d) No description of the derivation of the cohort

2) Ascertainment of exposure (to factors investigated in the studies)

a) Secure record (eg surgical records)

b) Structured interview

c) Written self-report

d) No description

3) Demonstration that retention/cessation (outcome of interest) is clearly defined

a) Yes
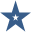


b) No

**Comparability**

1) Comparability of cohorts on the basis of the design or analysis (list factors adjusted for)

a) Multivariate analysis and factors adjusted for stated
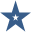


b) Multivariate analysis, no statement on factors adjusted for

c) Univariate analysis

d) Other analysis

**Outcome**

1) Assessment of outcome

a) Independent blind assessment

b) Record linkage

c) Self-report

d) No description

2) Was follow-up long enough for outcomes to occur (list timeframe of **follow**-**up**)

a) Yes (>6 months)

b) No (<6 months)

3) Adequacy of follow up of cohorts

a) Complete follow up - all subjects accounted for/ databases used

b) Subjects lost to follow-up unlikely to introduce bias - small number lost (<20%) and description provided of those lost)

c) Follow-up rate < 80% and no description of those lost

d) No statement
