# Supplementary material for: Retention of patients in opioid substitution treatment: A systematic review
Source: PLoS One. 2020 May 14;15(5):e0232086. doi: 10.1371/journal.pone.0232086 (PMC7224511; doi:10.1371/journal.pone.0232086)
Supplement: S5 Table — (DOCX) [file pone.0232086.s005.docx]

## S5 Table. Characteristics of included studies

| **Author** | **Country and**  **Treatment**  **Setting** | **Participants:**  **N**  **Sex**  **Mean age (±SD)**  **Age Range** | **OST type*** | **Intervention** | **Follow-up period** | **Primary outcome assessed** | **Study outcome(s) definitions** | **Treatment effects** |
| --- | --- | --- | --- | --- | --- | --- | --- | --- |
| Chawarski 2011(31) | 2 MMT Clinics China | N=37  81% men  36.7 years (7.2) | MMT | RCT: MMT only vs. MMT plus weekly individual drug and HIV counselling | 6 months | Retention | Retention defined as continuing to receive MMT at 6 months | No difference in retention: 83.3% retention in MMT only vs. 76.2% in MMT plus weekly counselling |
| Jaffray 2014(32) | 76 Community pharmacies, Scotland | N=542  64% men  32 years | MMT | Cluster RCT: Pharmacists randomised to intervention (Motivational interviewing training + resource pack); control ( usual care) | 6 months | Retention | A patient was considered retained if they were still receiving treatment from the same or another pharmacy or clinic. | No difference in retention : 88% retention in MMT in intervention group vs 81% in usual care |
| Marsch 2014(33) | Large MMT clinic, urban Northeastern USA | N=160  75% men  40.7 years (9.8) | MMT  New entries to MMT | RCT: MMT + counselling vs MMT +reduced counselling + web-based therapeutic education | 12 months | Retention | Retention calculated as the number of days each participant actively participated in study | No difference in retention: HR 0.94, p=0.74 |
| Schwartz 2012(34) | Two methadone programmes in Baltimore, USA | N=230  70% men  43.2 years (8.0) | MMT  New entries to MMT | RCT: Interim methadone (IM) (supervised methadone with emergency counselling); standard methadone (SM) (usual MMT with routine counselling); Restored methadone (RM) (routine counselling with smaller caseloads) | 12 months | Retention | Retention in treatment at 12 months | 55.2% retained at 12 months; no differences between the 3 groups in rates of retention (60.6% IM; 54.8% SM; 37% RM) |

| **Author** | **Country and**  **Treatment**  **Setting** | **Participants:**  **N**  **Sex**  **Mean age (±SD)**  **Age Range** | **OST type*** | **Study Design** | **Data collection / Follow-up period** | **Primary outcome assessed** | **Study outcome(s) definitions** |
| --- | --- | --- | --- | --- | --- | --- | --- |
|  |  |  |  |  |  |  |  |
| Abramsohn 2009 (35) | Israel, Tel Aviv  MMT clinic | N=90  73.3% men  39.8(±9.9) years  NR | MMT | Prospective cohort study | September 2003 -April 2006 | Retention | Duration in treatment from admission until the patient withdrew or until the end of follow-up (July 2007) was used for calculating cumulative retention in treatment. |
| Adelson 2013 (36) | Macao (Special Administrative Region of the People's Republic of China)  MMT clinic | N=163  81% men  39.5 (±10.2) years  Range: 20.4−71.8 | MMT | Prospective cohort study | October 2005 - March 2010 | Retention | One-year treatment retention defined as patient staying in treatment for a period of at least 1 year. Duration of treatment defined as starting at the time of patients’ initial admission and ending when patient either left treatment or until study end.  Long term retention (4.5 years) |
| Amiri 2018 (37) | USA, Washington  State funded opioid treatment program (clinic) | N = 851  49% men  36.3 (± 11.7) years  (NR) | MMT | Retrospective cohort study | February 2015 – December 2017 | Dropout | Dropout: time based on number of days in treatment from the first dose date until the client dropped out of treatment or the closure of the study on 31st December 2017; and status indicator to distinguish between those who dropped out or were censored. Clients who discontinued their treatment were coded as dropped out of treatment. |
| Astals 2009 (38) | Spain, Barcelona  1 MMT clinic | N=189  77% male  34(±7.5) years  NR | MMT | Prospective cohort study | NR | Dropout | Retention in the MMT at 18 months: remaining in the same MMT program and stable doses |
| Banta-Green 2009 (39) | USA, Washington State  11 MMT clinics | N=2308  51.6% men  40.6(±10.3) years  NR | MMT | Retrospective cohort study | January 2004 – December 2005 | Retention | 12 month retention: remaining in treatment at day 366 following admission to MMT. |
| Bhatraju 2017 (40) | USA, New York City  Primary Care Centre, Office based buprenorphine treatment in a large urban-public hospital | N=485  83% men  47 (SD NR) years  Range: 23–73 | BUP. | Prospective cohort study | August 2006 -June 2013 | Dropout | Treatment retention: continuous variable (weeks) consisting of the time between the initial and last week of the last active buprenorphine maintenance or taper prescription. Dropout defined as time to dropout |
| Bounes 2013 (41) | Mixed: Emergency departments, surgery departments, specialised addiction centres, France | N=151  74% men  Median age:36years  Range:20-54 years | Mixed OST | Prospective cohort study | April 2008-January 2010 | Retention | Retention defined as the percentage of patients still under treatment at the time of follow-up  Lost to follow-up patients were analysed as failure |
| Brands 2008(42) | Canada, Centre for Addiction and Mental Health (CAMH) | N=172  64% male  34.6 years (8.5)  NR | MMT | Retrospective cohort study | January 1997-December 1999 | Dropout | Retention: only those patients who discontinued methadone counted as discharged; those who were transferred to another MMT treatment programme considered in treatment |
| Bukten 2014 (43) | Norway  National cohort | N=2431  67% men  Men  37.8(±6.5) years  Range: 22–60  Women  35.5(±6.3) years  Range: 20-57 | Mixed OST | Retrospective cohort study | September 1997 -December 2003 | Dropout | Treatment dropout defined as the first day of the period of discontinuing treatment. Termination of treatment could be either voluntary or involuntary. |
| Burns 2009 (44) | Australia, New South Wales  Multiple settings: clinic; correctional facilities; community pharmacy; public hospital; general practitioners; other | N=42,690  **1986** 61.1% men  Median age: 27.5  **1991** 65.4% men  Median age: 28.3  **1996** 68.8% men  Median age: 26.4  **2001** 64.7% men  Median age 26.5  **2006** 71.3%  Median age: 28.2 | Mixed OST | Retrospective cohort study | 1985 - 2006 | Dropout | An episode of continuous treatment as one with no more than a 6-day break between treatment programmes. Where a gap of 7 days or more occurred between an exit date and a start date, a new episode of treatment was defined.  Dropout from first treatment episode was measured as date of leaving first treatment episode |
| Cao 2014 (45) | First 8 MMT clinics in China | N=1511  77.1% men  Age:  ≥30 years = 60.3%  NR | MMT | Prospective cohort study | March 2004 - June 2010 | Dropout | Retention: Clients who were still on methadone at the end of the study period (June 2010) considered to be retained over the six-year period.  Retention duration was calculated from the date of first methadone treatment to the date of the last methadone treatment or the study end date, whichever occurred first. |
| Cox 2013 (46) | Canada  Cape Breton Methadone Maintenance Program | N=246  68% men  31 (SD NR) years  NR | MMT | Retrospective cohort study | May 2004 –  May 2007 | Dropout | Dropout defined as loss to follow-up: includes involuntarily discharged from the program or if they voluntarily interrupted (voluntary discharge) methadone therapy for more than 7 days. Involuntarily discharge: if they were aggressive towards clinic staff, or if they continued to use opioid analgesics or other non-prescribed substances despite numerous attempts to stay within the program.  Retention: those who were active in the program as of May 1, 2007, who completed the program (patients who were stabilized on and voluntarily tapered from methadone) and those who had transferred. |
| Cunningham 2013 (47) | USA, Bronx community health centre  OBOT | N=87  73.6% men  43.5(± 9) years  NR | BUP. | Prospective cohort study | November 2004 -  December 2009 | Retention | Treatment retention at 6 months after participants initiated buprenorphine treatment. Participants were categorized as retained in treatment if they had either a medical visit or active buprenorphine prescription. To be considered as retained at 6 months, participants had to be retained at both 1 and 3 months |
| Davstad 2007 (48) | Sweden  MMT clinic | N=204  72% men  Men N=147  Age range: 21-66  Women N=57  Age range: 24-50 | MMT | Prospective cohort study | 1995 - 2000 | Dropout | Retention in treatment at 1 year and 2 years.  Subjects in treatment on 31^st^ Dec 2000 are retained |
| Dayal 2017 (49) | Drug treatment centre, India | N=68  NR  22.35(±2.47) years  NR | BUP. | Retrospective cohort study | January 2012 –  March 2015 | Dropout | Discontinuation of buprenorphine maintenance treatment was defined as 7 consecutive days without buprenorphine or buprenorphine-naloxone.  Duration of time from initiation to discontinuation was time, measured in days, from first prescription to last prescription (including day’s supply of last prescription) |
| Deck 2005 (50) | USA, Oregon and Washington  MMT clinics | N=5308  45% men  38.9 (SD NR) years  NR | MMT | Retrospective cohort study | 1994- 1999 (2000) | Retention | Retention was defined as continuous enrolment in an index treatment episode with no discharge for at least 12 months following the admission date. The retention rates were calculated as the number of individuals retained in the MMT for 12 months divided by the number of individuals admitted to the MMT during the same period. The rates were expressed as percentages. |
| Dumchev 2017 (51) | Ukraine  13 OAT clinics | N=2916  77.9% men  36.4 (7.9) years  Range: 18–70 | Mixed OST | Retrospective cohort study | 2005 -2012 | Dropout | 12 month retention in treatment, defined as the time between treatment initiation and discontinuing treatment for 10 or more consecutive days. Time to dropout using date of admission and date of discharge |
| Eibl 2015 (52) | Canada, Ontario  MMT clinics | N=17211  44-50% male  Median age 35 – 39  NR | Mixed OST | Retrospective cohort study | 2003 - 2012 | Retention | Retention: defined a patient as having been retained in treatment if they completed at least one year of continuous uninterrupted treatment (i.e. no period of 30 consecutive days without a prescribed dose of methadone or buprenorphine) |
| Franklyn 2017 (53) | Canada, Province of Ontario  58 OAT clinics | N=3850  60% men  Median age 31.4 years | Mixed OST | Retrospective cohort study | January 2011 - June 2012 | Dropout | Retention: a patient was defined as retained in treatment if they completed at least for one year of continuous and uninterrupted treatment (continuous treatment on the basis of not having a period of 30 or more consecutive days without a dose of methadone or buprenorphine/naloxone)  Dropout: treatment dropout (patient did not receive methadone or buprenorphine/naloxone dose for 30 consecutive days) |
| Friedmann 2001 (54) | USA  22 MMT outpatient clinics from Drug Abuse Treatment Outcome Study (DATOS) | Partial sample (outpatient MMT)  N = 1144  60.7% male  36.2 (±7.0) years  NR | MMT | Retrospective cohort study | 1991 - 1993 | Retention | Time (days) each client spent in treatment was found by subtracting the date of admission from date of last therapeutic contact based on information in client records. The treatment retention variable was dichotomized at retention threshold 365 days or more in treatment |
| Gerra 2011(55) | Italy – 3 MMT Clinics with different policies for MMT (A –strictly supervised daily consumption 6 days a week; take-home methadone on Sunday; B- take home methadone in a behavioural/incentive perspective; C- early non-contingent take-home methadone | N=300 (100 per clinic)  A 82%, B 84%, C 81% male  A (28.19); B (27.9); C (28.77)  NR | MMT | Prospective Cohort Study | June 2006-June 2008 | Dropout | Retention in treatment (checked every few weeks); dropout after 12 months |
| Gryczynski 2014 (56) | Baltimore USA  African Americans attending 2 outpatient programmes | N=297  61.9% men  46.6 years (6.5)  NR | BUP. | Secondary analysis of RCT data | March 2010-March 2011 | Dropout | Retention: Number of days in treatment from clinic records, 1-180 days, after which observations censored. |
| Gu 2012 (57) | China, Guangdong  3 MMT clinics | N=158  90.5% men  Age:  < 35 years = 26.5%  36-40 years = 40%  40+years =33.5% | MMT | Prospective cohort study | May 2009 - October 2010 | Dropout | Dropout was defined as not having visited the MMT clinic for at least 1 month prior to the study’s completion date. |
| Haddad 2013(58) | USA, Connecticut, Community Health Centre, 2 sites | N=266  69.2% men  40.1 years (NR)  20-64 | BUP. | Retrospective cohort study | July 2007-December 2008 | Dropout | Retention was defined as being on BMT at the end of the pre-specified time period, it was defined as the time until initial discontinuation of BMT |
| Huissoud 2012 (59) | Switzerland, Canton of Vaud | N=1666  71.4% men  Age:  ≥30 years = 42%  ≤ 30 years = 57.9% | MMT | Retrospective cohort study | January 2001 - June 2008 | Dropout | Dropout defined as ending treatment and included the following reason, dropping out, methadone withdrawal, transfer, or entry to prison |
| Johns 2018 (60) | Vietnam, 7 provinces  MMT clinics | N = 1,021  No user fee policy  99% men  Mean age 37.7 years  User fee policy  96% men  Mean age 34.7years  NR | MMT | Prospective cohort study | May 2015-June 2016 | Dropout | Dropout: when patient has not come for MMT for 30 consecutive days and no reason for lack of attendance given to or ascertained by facility staff. Did not include those who stopped MMT with staff permission, and unclear if excluded those who failed a urine test |
| Kayman 2006 (61) | USA, New York  Hospital-affiliated MMT clinic | N=338  75% men  39(±8.33) years  NR | MMT | Prospective cohort study | July 1997 -  May 1999 | Dropout | Treatment provider provided weekly updates on list of patients who had left treatment. This detail used to identify those who had dropped out within a year after enrolment |
| Kelly 2011 (62) | USA, Baltimore, 6 MMT clinics | N=351  53.3% men  41.2(±8.2) years  NR | MMT | Prospective cohort study | November 2004 – November 2007 | Dropout | Retention: number of days enrolled in the index treatment program through 12 months (maximum number of days = 365). |
| Lambdin 2014 (63) | National Hospital Tanzania | N=629  93% men  32(±6) years  NR | MMT | Retrospective cohort study | February 2011 - January 2013 | Dropout | Dropout: Attrition, dropping out of MMT defined as 21 consecutive missed doses, and a client’s last pharmacy refill was assigned as the date of dropout. |
| Ledgerwood 2019 (64) | USA, Detroit  University affiliated urban MMT clinic | N = 290  Non Injector (n=175)  57.1% men  Age : ≥55 years - 35.4%  NR  Injector (N= 115)  63.5% men  Age : ≥55 years – 30.4%  NR | MMT | Retrospective cohort study | 2002 - 2009 | Retention | Retention: Number of days retained in MMT |
| Lin 2013 (65) | Taiwan, 3 Hospital based outpatient clinics | N=368  86.4% men  37.2(±7.6) years  NR | MMT | Prospective cohort study | Recruited March 2007 –  July 2008 with 18 month follow-up | Dropout | Dropout at 18 months. Duration of retention was defined as entry into MMT to discontinuation of the treatment |
| Lin 2015 (66) | Taiwan, 4 MMT outpatient clinics | N = 128  88.3% men  mean age 36.65 ± 8.34 years  NR | MMT | Prospective cohort study | July 2008 – April 2010 | Dropout | Patients had to go daily to clinic to receive methadone dose, and were considered treatment dropouts if they failed to take their methadone for 14 consecutive days |
| Liu 2017 (67) | China, Guangzhou  4 MMT Clinics | N=401  87.5% men  Age:  < 40 years = 29.2%  ≥ 40 years =70.8% | MMT | Prospective cohort study | August 2013 - August 2014 | Dropout | Dropout was defined as not having visited the clinic for at least 30 consecutive days prior to the study’s completion date. |
| Manhapra 2017 (68) | USA, National Veterans Health administration records | N = 3,151  94.1% men  42.8 years | BUP. | Retrospective cohort study | October 2011 - September 2015 | Dropout | Dropout: Duration of treatment calculated from first day of BUP filled to last day BUP was filled from 2011 to 2015 |
| Manhapra 2018 (69) | USA, Nationwide study of insured individuals of large employers and private health plans using the MarketScan Commercial Claims and Encounters Database. | N = 16,190  65.2% men  NR | BUP. | Retrospective cohort study | Federal fiscal (FY) year 2011 – federal fiscal year 2014 | Dropout | Treatment discontinuation at 3 years – time to discontinuation based on date of first day of BUP or BUP/naloxone fill in FY 2011 and last date BUP or BUP/naloxone was filled (up to FY 2014) |
| Meshberg – Cohen 2018 (70) | Veterans enrolled in BUP treatment at Veteran Affairs (VA) Connecticut Healthcare System USA | N = 140  92.9% men  45.4 (± 12.7) years  NR | BUP. | Retrospective cohort study | October 2015 – March 2017 | Retention | Retention: defined as sustained treatment engagement through the 6-months post-admission period as evidenced by BUP clinic attendance, pharmacy pickups, prescription renewals and no note indicating treatment dropout |
| Monico 2015 (71) | USA,  Maryland  Two outpatient programs | N=300  62% men  46 (±6.45) years  NR | BUP. | Secondary analysis of a randomised control trial | NR | Retention | Retention: in treatment at 6 months. Not reported how this was ascertained (combination of self-report and clinic records) |
| Montalvo 2019 (72) | USA, Boston  Buprenorphine clinic | N = 321  62% men  38 (± 10) years  NR | BUP. | Retrospective cohort study | January 2010 – February 2016 | Retention | Retention: continuous treatment with buprenorphine for greater than or equal to one year. The duration of patients’ continuous treatment episodes was determined from the elapsed time between patients’ initial buprenorphine prescription and the last day of an active prescription before disengaging from the treatment. Longest episode is selected |
| Mullen 2012 (73) | Ireland  National treatment register (specialist drug treatment centres; Community drug treatment centres; primary care setting) | N=1269  68.6% men  26 (SD NR) years  NR | MMT | Retrospective cohort study | 1999, 2001, 2003 | Retention | Retention in treatment at 12 months, based on entry and exit dates in national register |
| Nosyk 2009(74) | British Columbia PharmaNet database all persons receiving MMT | N=17,005  66% male  65% aged 20-40 years  NR | MMT | Retrospective cohort study | January 1996-December 2006 | Dropout | Time to discontinuation: the length of a treatment episode was calculated as the difference between the last and first days of medication dispensed, within a period continuous retention in treatment, where continuous entailed no interruptions in prescribed doses lasting longer than 30 days |
| Peles 2008 (75) | USA (Nevada) and Israel (Tel Aviv)  2 MMT clinics | N=704  **Tel Aviv (492)** :  72.8% men  36.7(±8.5) years  Range: 18-67  **Las Vegas (302):**  62.9% men  43.4(±9.4) years  Range:19-63 | MMT | Prospective cohort study | Las Vegas:  February 2000 –  April 2005  Tel-Aviv:  June 1993 –  June 2004 | Retention | 6 months retention, 1 year retention in treatment, and the total duration in treatment (dates of admission and date of discharge if a patient left treatment or date of end of follow-up if a patient was still in treatment for computing cumulative retention). |
| Peles 2018 (76) | Tel Aviv, Israel  University affiliated clinic | N = 890  74.8% men  Age  18-39 years – 53.6%  ≥ 40 years – 46.4%  NR | MMT | Retrospective cohort study | June 1993 – June 2017 | Retention | Retention: first MMT admission until the patient died, left, or until the end of follow-up. |
| Perreault 2015(77) | Canada, Montreal  Low threshold programme | N=106  74.5% men  28.5 (± 8.6) years  Range: 18 to 59 | MMT | Prospective cohort study | 2001 - 2006 | Retention | Treatment retention defined as (i) being treated in the methadone program at the 1-year follow-up period or (ii) having been transferred to conventional “regular” maintenance programs or to private physicians.  Participants were considered dropouts if they did not receive methadone treatment for a month within the treatment service offered. |
| Perreault 2005 (78) | Canada, Montreal  Low threshold clinic | N=141  51% men  26.8(±6.5) years  NR | MMT | Prospective cohort study | November 1999 - October 2000 | Dropout | At the end of 6 month follow-up, clients categorised at treatment failure or treatment success. Failure included dropout (clients who did not go to the pharmacy for their dose of methadone for 7 consecutive days), and administrative discharges (discharged due to violent or antisocial behaviour); Treatment success included clients who were still in treatment at end of follow-up period |
| Proctor 2015 (79) | USA  26 MMT clinics | N=1644  63.1% men  34.7 (± 11.06) years  NR | MMT | Retrospective cohort study | January 2009 - April 2011 | Dropout | Dropout: premature treatment discharge at the 6-month (length of treatment ≤179 days) and 12-month (≤364) follow-up interval. |
| Ren 2013 (80) | China, Shanghai  MMT clinics | N=2463  77.8% men  40.9(±8.5) years  NR | MMT | Retrospective cohort study | January 2007 - December 2011 | Dropout | Dropout: an event with more than 30 suspended days.  Suspended days: a period of time that did not have methadone dosing records continuously recorded, except if there was a temporary or permanent referral to other provinces. |
| Ruadze 2016 (81) | Georgia  MMT clinics | N=1051  97.2% men  40.4 (SD NR) years  21 -67 years | MMT | Retrospective cohort study | 2014 - 2015 | Retention | Retention: (1) more than 6-months retention (≥181 days stay); (2) more than 9 months retention (≥270 days stay); (3) more than 12 months retention (≥365 days stay) |
| Saloner 2017 (82) | USA, 11 states  Community based | N=27,273  52% men  NR | BUP. | Retrospective cohort study | January 2010 - July 2012 | Retention | 6 month retention (binary measure of treatment episodes 180 days or longer) After 90 days of no fills the episode was defined as terminated |
| Sarasvita 2012 (83) | Indonesia  3 MMT clinics - | N=178  90% men  27.2(±4.8) years  NR | MMT | Prospective cohort study | July 2006 – January 2008 | Dropout | Retention: The duration of treatment (in days). Study participants who left treatment but subsequently re-entered within 5 days were counted as a continuous episode of treatment. Participants who re-entered after 5 days had the subsequent episode counted as a separate treatment episode.  Dropping out of treatment was defined as a participant failing to take a daily dosage of methadone for a minimum of five consecutive days |
| Schuman-Olivier 2013 (84) | USA, Boston  OBOT | N=328  59.7% men  No BZD treatment N=270  64.8% men  35.6 (±10.5) years  NR  BZD treatment N=58  36.2% men  37.7 (± 11.6) years  NR | BUP. | Retrospective cohort study | November 2007 - June 2010 | Retention | 12-month treatment retention. |
| Shakira 2017 (85) | Malaysia, Khota Bharu  Tertiary hospital MMT programme | N = 178  98.3 % men  31.5 (± 5.2) years  (18-58) | MMT | Retrospective cohort study | November 2005 – November 2010 | Dropout | Dropout: failure of retention that includes voluntary dropout or involuntary dropout (due to excluded, imprisoned, transferred to other MMT or death due to any cause) |
| Shcherbakova2018 (86) | USA, Massachusetts  Insurance claims data from a regional health plan  Employer – sponsored and Medicaid patients | N = 302  71.5% men  34.5 (± 12.7) years  NR | Mixed OST | Retrospective cohort study | June 2010 – January 2015 | Retention | Retention (treatment persistence): The number of days from the fill date of the first buprenorphine containing prescription until more than 30 consecutive days without buprenorphine. |
| Socias 2018 (87) | Canada, Vancouver  VIDUS and ACCESS cohorts | N = 820  57.8% men  Median age: 38 years (no SD)  NR | Mixed OST | Prospective cohort study | December 1996 –May 2016 | Retention | Retention: Defined as a self-report of being on methadone or buprenorphine/naloxone based treatment in the current and immediately previous follow-up interview (approximately a 6 month retention interval) |
| Stein 2005 (88) | USA, Rhode Island  Primary care | N=41  58.5% men  40.1 (±7.83) years  NR | BUP. | Prospective cohort study | 2003 | Dropout | Dropout not defined |
| Strike 2005 (89) | Canada  Population based MMT treatment register | N = NR  9555 treatment episodes  70.3% men  34.9 (SD NR) years  NR | MMT | Retrospective cohort study | January 1996 - December 2001 | Retention | 2-year retention (730 days), an interval of greater than 7 days between a cessation and a new MMT registration constituted cessation of a treatment episode |
| Sullivan 2013 (90) | China  540 MMT clinics | N=107,740  85% men  35 (SD NR) years  NR | MMT | Retrospective cohort study | April 2008 - March 2010 | Retention | Duration of treatment: follow-up began on the date of entry, on or later than 1^st^ April 2008, and ended when a client missed 30 days or more or at study end (31 march 2010) |
| Teoh 2017 (91) | Malaysia, Kuala Lumpur  Tertiary hospital MMT programme | N = 164  100% men  40.7 (± 10.1) years  NR | MMT | Retrospective cohort study | 2005 – 2015 | Dropout | Retention: time of entry to the MMT programme either until dropout or June 2015. |
| Wei 2013(92) | Xi China, 8 MMT Clinics | N=5849  84.1% men  NR | MMT | Retrospective cohort study | January 2006-December 2011 | Dropout | Retention: Number of days from admission until the patient quit treatment or until the end of follow-up. End date Dec 2011, before this date, patients who were absent from MMT for 30 days were considered to have dropped out |
| Weinstein 2017 (93) | USA, Boston  Office based opioid treatment | N=1237  61.4% men  38 (± 11) years  NR | BUP. | Retrospective cohort study | January 2002 - February 2014 | Retention | Retention defined as at least one year of continuous treatment with buprenorphine (individual was in treatment for at least 365 days, as long as any gap in care was <60 days).  “≥2 year retention” was also explored |
| Yang 2013 (94) | China, Guangdong  6 MMT clinics | N=2728  72.8% men  36.4(±12.4) years  NR | MMT | Retrospective cohort study | January 2006 - September 2010 | Dropout | Retention: patients remaining in MMT along the period of study (January 1st, 2006 and September 30th, 2010) or who were temporarily referred to other MMT clinics other than the selected 6 clinics.  Retention duration is calculated from first MMT entry and up to the date patients dropped out of treatment or the end of the follow-up period (September 30th, 2010). |
| Zhang 2015 (95) | China, Guangdong Province,  14 MMT clinics | N=1512  90.0% men,  38.6 (5.99) years  Range: 21–63 | MMT | Ambi-directional cohort study | 2006 - 2014 | Dropout | An individual was considered as ‘dropout’ if they missed methadone intake for ≥14 consecutive days. Participants were considered as “ever dropouts” if only he/she dropout in their initial treatments or in the subsequent period of time during the study duration.  Participants who did not dropout were considered ‘retained’. |
| Zhou 2017 (96) | China, Xi'an  2 MMT clinics, one public one private | N=1212  77.1% men  39.24 (± 6.24) years  Range: 19-69 | MMT | Prospective cohort study | March 2012 - March 2014 | Dropout | Retention defined as premature terminations, reflecting whether patients continued MMT after the 2-year follow-up; patients who were off methadone for 7 consecutive days were considered to have terminated. |
| Zhou 2016 (97) | China, Xi'an  14 MMT clinics | N=10398  83.9% men  38.06 (± 6.98) years  Range: 17-69 | MMT | Retrospective cohort study | January 2006 - December 2013 | Dropout | Dropout: patients with a difference of >7 days between the date of last methadone intake and the endpoint identified as the ‘‘dropout’’ group; patients with a difference of ≤7 days between the date of last methadone intake and the endpoint were defined as the ‘‘retained’’ group. |

*****MMT = Methadone maintenance treatment; BUP. = Buprenorphine or Buprenorphine-naloxone

N = Number of participants; NR = Not Reported; OAT = Opioid antagonist treatment; OST = Opioid Substitution Treatment; OBOT = Office Based Opioid Treatment (outpatient treatment);

SD = Standard Deviation; USA = United States of America;
