# Supplementary material for: Retention of patients in opioid substitution treatment: A systematic review
Source: PLoS One. 2020 May 14;15(5):e0232086. doi: 10.1371/journal.pone.0232086 (PMC7224511; doi:10.1371/journal.pone.0232086)
Supplement: S6 Table — (DOCX) [file pone.0232086.s006.docx]

## S6 Table. Results of the critical appraisal of included observational studies using the Newcastle Ottawa Scale (n=63)

|  | **Selection** | | | **Comparability** | **Outcome** | | |  |
| --- | --- | --- | --- | --- | --- | --- | --- | --- |
| **Study** | **Representativeness of sample** | **Ascertainment of Exposure** | **Definition of outcome** | **Comparability** | **Ascertainment of outcome** | **Duration of follow-up** | **Adequacy of follow-up** | **Study total out of 7 stars:** |
| Abramsohn 2009 (35) |  |  | * |  | * | * | * | **4** |
| Adelson 2013 (36) |  | * | * | * | * | * | * | **6** |
| Amiri 2018 (37) |  | * |  | * | * | * | * | **5** |
| Astals 2009 (38) |  | * | * | * | * | * | * | **6** |
| Banta-Green 2009 (39) | * | * | * | * | * | * | * | **7** |
| Bhatraju 2017 (40) |  | * |  | * | * | * | * | **5** |
| Bounes 2013 (41) | * | * | * | * | * | * | * | **7** |
| Brands 2008(42) |  | * | * | * | * | * | * | **6** |
| Bukten 2014 (43) | * | * |  | * | * | * | * | **6** |
| Burns 2009 (44) | * | * | * | * | * | * | * | **7** |
| Cao 2014 (45) | * | * | * | * | * | * | * | **7** |
| Cox 2013 (46) |  | * | * | * | * | * | * | **6** |
| Cunningham 2013 (47) |  | * | * | * | * | * | * | **6** |
| Davstad 2007 (48) | * | * | * |  | * | * | * | **6** |
| Dayal 2017 (49) |  | * | * | * | * | * | * | **6** |
| Deck 2005 (50) | * | * | * | * | * | * | * | **7** |
| Dumchev 2017 (51) | * | * | * | * | * | * | * | **7** |
| Eibl 2015 (52) | * | * | * | * | * | * | * | **7** |
| Franklyn 2017 (53) | * | * | * | * | * | * | * | **7** |
| Friedmann 2001 (54) | * | * | * | * | * | * | * | **7** |
| Gerra 2011(55) | * | * |  |  | * | * | * | **5** |
| Gryczynski 2014(56) | * | * |  |  | * | * | * | **5** |
| Gu 2012 (57) | * | * | * | * | * | * | * | **7** |
| Haddad 2013(58) | * | * | * | * | * | * | * | **7** |
| Huissoud 2012 (59) | * | * |  | * | * | * | * | **6** |
| Johns 2018 (60) | * | * | * | * | * | * | * | **7** |
| Kayman 2006 (61) |  | * | * | * | * | * | * | **6** |
| Kelly 2011 (62) |  | * |  |  | * | * | * | **4** |
| Lambdin 2014 (63) |  | * | * | * | * | * | * | **6** |
| Ledgerwood 2019 (64) |  |  | * | * | * | * | * | **5** |
| Lin 2013 (65) |  | * |  | * | * | * | * | **5** |
| Lin 2015 (66) | * | * | * | * | * | * | * | **7** |
| Liu 2017 (67) | * | * | * | * | * | * | * | **7** |
| Manhapra 2017 (68) |  | * |  | * | * | * | * | **5** |
| Manhapra 2018 (69) |  | * |  | * | * | * | * | **5** |
| Meshberg – Cohen 2018 (70) |  | * | * | * | * | * | * | **6** |
| Monico 2015 (71) |  | * | * | * |  | * | * | **5** |
| Montalvo 2019 (72) |  | * | * | * | * | * | * | **6** |
| Mullen 2012(73) | * | * | * | * | * | * | * | **7** |
| Nosyk 2009(74) | * | * | * | * | * | * | * | **7** |
| Peles 2008 (75) |  | * | * | * | * | * | * | **6** |
| Peles 2018 (76) |  | * |  | * | * | * | * | **5** |
| Perreault 2005(77) |  | * | * | * | * | * | * | **6** |
| Perreault 2015(78) |  | * | * | * | * | * | * | **6** |
| Proctor 2015 (79) |  | * | * | * | * | * | * | **6** |
| Ren 2013 (80) | * | * | * | * | * | * | * | **7** |
| Ruadze 2016 (81) | * | * |  | * |  | * | * | **5** |
| Saloner 2017(82) | * | * | * | * | * | * | * | **7** |
| Sarasvita 2012 (83) | * |  | * | * | * | * | * | **6** |
| Schuman-Olivier 2013 (84) | * | * |  | * |  | * | * | **5** |
| Shakira 2017 (85) |  |  |  | * | * | * | * | **4** |
| Shcherbakova 2018 (86) | * | * | * | * | * | * | * | **7** |
| Socias 2018 (87) |  | * | * | * |  | * | * | **5** |
| Stein 2005 (88) |  | * |  |  | * | * | * | **4** |
| Strike 2005 (89) | * | * | * | * | * | * | * | **7** |
| Sullivan 2013(90) | * | * | * | * | * | * | * | **7** |
| Teoh 2017 (91) |  | * |  | * | * | * | * | **5** |
| Wei 2013(92) | * | * | * | * | * | * | * | **7** |
| Weinstein 2017 (93) |  | * | * | * | * | * | * | **6** |
| Yang 2013 (94) | * | * |  | * | * | * | * | **6** |
| Zhang 2015 (95) | * | * | * | * | * | * | * | **7** |
| Zhou 2017 (96) | * | * | * | * |  | * | * | **6** |
| Zhou 2017 (97) | * | * | * | * | * | * | * | **7** |
|  |  |  |  |  |  |  |  |  |
