# Supplementary material for: Retention of patients in opioid substitution treatment: A systematic review
Source: PLoS One. 2020 May 14;15(5):e0232086. doi: 10.1371/journal.pone.0232086 (PMC7224511; doi:10.1371/journal.pone.0232086)
Supplement: S7 Table — (DOCX) [file pone.0232086.s007.docx]

## S7 Table. Summary of results of included studies; potential risk and protective factors explored

| **First author** | **Retention**  **or**  **Dropout**  **Rates** | **Outcome**  **Model and coefficients reported** | **Demographics**  **Age**  **Gender**  **Location**  **Race** | **Substance use**  **Poly-drug use**  **Cannabis use**  **BZD use**  **Cocaine use**  **Heroin use**  **Alcohol use**  **Amphetamines/ecstasy**  **Drug use patterns** | **Treatment Factors**  **Medication type**  **Dosage**  **Previous treatments**  **Year of treatment intake**  **Treatment setting**  **Treatment facilities** | **Health Risk Behaviour**  **sexual behaviour**  **Injecting**  **Sharing needles** | **Health Symptoms**  **Non-fatal overdose**  **Clinical diagnosis:**  **Mental health (self-reported)**  **Physical health (self-reported)**  **HIV+/-**  **TB +/-** | **Social functioning**  **Marital Status**  **Employment status**  **Income**  **Education**  **Living status**  **Social Support**  **Legal issues** | **Other variables investigated in included studies** |
| --- | --- | --- | --- | --- | --- | --- | --- | --- | --- |
| Abramsohn 2009 (35) | Retention at 1 year (85.6%) | Retention at 1 year  Survival analysis with log rank of retention |  |  | - | - | - | - | High (>130) Sense of Coherence score – S  3.50 (3.20-3.90) years  Low (≤130) Sense of Coherence score – S  2.90(2.50-3.30) years |
| Adelson 2013 (36) | Retention at 1 year (59.5%) | Long-term treatment retention (4.5 years)  Cox regression model  Hazard Ratios (95% CI) | - | - | Methadone dose (high- ≥80mg/day) – S  2.20 (1.30-3.70) | - | Being sera positive to hepatitis C on admission – S  1.80 (1.10–2.80) | - | - |
| Amiri 2018 (37) | Retention at 1 year (50%); at 3 years (30%) | Dropout (time to drop out between 2015-2017)  Cox proportional hazard model (95% CI)  Hazard Ratios (95% CI) | Age – S  0.99 (0.98 - 0.99)  Gender (ref: female) – NS  1.17 (0.96 - 1 .41)  Area deprivation index :  (ref: Least deprived < 100)  Most deprived > 100 – S  1.79 (1.02 – 3.15)  Ethnicity (ref: non-Hispanic white) other – NS  1.00 (0.79 – 1.26) |  | Methadone dose (mean, SD) – S  0.98 (0.98 – 0.98)  Years on treatment (median, IQR) – S  1.12 (1.06 – 1.18) |  |  | Cannabis retail outlets in neighbourhood (median) – NS  1.08 (0.99-1.18)  Off-premise alcohol outlets in neighbourhood (median) – NS  0.99 (0.96-1.03)  On-premise alcohol outlets in neighbourhood (median) – NS  1.00 (0.99-1.00) | Distance to OTP (ref: < 5 miles):  >5 miles and <10 miles – NS  0.84 (0.65 – 1.09)  > 10 miles – NS  1.12 (0.82 – 1.54) |
| Astals 2009 (38) | Retention at 18 months (68.5%) | Dropout (follow-up 18 months)  Logistic regression  Odds Ratios (95% CI) | Age – NS  (no figures reported)  Male – NS  2.59 (0.98–6.84) | Current diagnosis of cocaine and opiates other than heroin dependence – NSU- | Methadone dose NSU | - | Co-occuring psychiatric disorders NSU | Lower educational level – NS  2.65 (0.98–7.13) | - |
| Banta-Green 2009 (39) | Retention at 12 months  Prescription type opioid only users (54%); Those who had used heroin (46%) | Retention at 12 months  Logistic regression  Odds Ratios (95% CI) | Age (10 year increments) – S  1.27(1.15-1.40)  Female – NS  1.12(0.93-1.34)  Black (ref: white) - S  0.69 (0.52-0.91)  Native American – NS  0.95(0.62-1.44)  Other race – NS  0.96(0.72-1.30) | Cocaine – S  0.75(0.63-0.91)  Methamphetamine – S  0.62(0.44-0.89) | In treatment for prescription-type opioid primary(vs. heroin use) – NS  1.25(0.93-1.67) |  | Clinical diagnosis:  Medical concerns – S  0.74(0.62-0.88) | Public assistance (ref: no public assistance):GAU(low income ,temporarily disabled): -S  1.58(1.19-2.09)  TANF(parenting) –S  1.89(1.37-2.60)  GAX or SSI (disabled) – S  1.86(1.48-2.35)  Medical assistance only – NS  1.06(0.71-1.59)Other - NS  1.42(0.37-5.46)Home conducive to recovery – S  1.52(1.26-1.84)  Children under 12 at home - S  1.35(1.03-1.78)  Legal system involvement – S  0.71(0.57-0.89) | Agency (ref: agency 1) : agency 2 – NS  0.57(0.30-1.08)  Agency 3 – NS  0.62(0.34-1.14)  Agency 4 – NS  0.90(0.57-1.43)  Agency 5 – NS  0.66(0.42-1.05)  Agency 6 – S  0.62(0.44-0.89)  Agency 7 – NS  0.82(0.56-1.20)  Agency 8 –S  0.73(0.53-0.99)  Agency 9 – NS  0.95(0.74-1.23)  Agency 10 – NS  1.15(0.79-1.66)  Agency 11 – NS  0.85(0.48-1.48) |
| Bhatraju 2017 (40) | Median retention 57 weeks | Dropout (Time to dropout between 2006 and 2013)  Cox proportional hazard models  Model 1: total sample; Model 2: Induction Patients  Hazard Ratios (95% CI) | Model 1 (total sample): Age - NS  0.98(0.97-1.00)Gender - NS  Ethnicity- NS  Model 2 (induction patients)  Age - NS  0.99 (0.97-1.00)  Gender - NS  Ethnicity- NS | Model 1 (total sample): Active Cocaine use - NS  1.18(0.90–1.54)  Active heroin use - NS 1.25(0.96–1.64)  Model 2 (induction patients)  Active Cocaine use - NS  1.15(0.83–1.59)  Active heroin use - NS 1.27(0.93–1.75) | Model 1 (total sample): Prior buprenorphine use – NSU  Year of first visit (ref: 2007):  2008 – NS  0.81 (0.54–1.20)  2009 –S  0.67 (0.49–0.91)  2010 - NS  0.75 (0.54–1.04)  2011 - NS  0.70 (0.49–1.01)  2012 - NS  0.61 (0.35–1.05)  2013 - NS 0.62 (0.29–1.34)  Inducted – NS:  1.46 (1.10–1.93)  Outpatient counselling active – NSU  12 step attendance active - NSU  Any induction related adverse effects - NSU  Methadone to buprenorphine induction - NSU  Model 2 (induction patients)  No factor significantly associated with time to dropout | - | - |  | - |
| Bounes 2013 (41) | Retention rate at 12 months (33%) | Retention at 12 months  Logistic Regression  Odds Ratio (95% CI) | Gender (ref Male)-NS  1.66 (0.71-3.87)  Age-(ref < 30 yrs)-NS  30-40 yrs: 1.08 (0.42-2.75)  >40 yrs: 1.56 (0.59-4.18) |  | Type of OST (BUP ref MMT)- S  0.33 (0.15-.072) |  | Acute pain exposure (ref unexposed)-NS  0.48 (0.23-1.00) |  |  |
| Brands 2008 (42) | Retention at 2 years (60%) | Dropout  Cox Regression analysis | Age-NS (NR)  Gender- NS (NR) | Opioid history-NS (NR)  Benzodiazepine-NS (NR)  Opioid and Cocaine use-S (β=0.95; p=0.008) HR 2.6 |  |  | Psychiatric treatment history-NS (NR) |  |  |
| Bukten 2014 (43) | 18 month retention rate (65.8%) | Dropout at 18 months  Cox proportional-hazards regression  Hazard Ratios (95% CI) | Female – NS  0.95(0.82-1.11)  Age – S  0.97(0.96-0.98) | - | - | - | - | Pre-treatment offences (ref: none):  Medium (1-24) offences – NS  1.03(0.87-1.22)  High (25+) offences - S  1.66(1.32-2.09)  Drug offences in the last 30 days – S  1.80(1.36-2.38) | - |
| Burns 2009 (44) | Retention  1985: retention at 6 months (59%); at 12 months (50%)  2000  Retention at 6 months (54%); at 12 months (41%) | Dropout in the first 9 months and after 9 months  Cox proportional hazards model of leaving the first treatment episode, splitting the follow-up at 9 months (1985-2000) and 6 months (2001-2006).  Hazard Ratios (95% CI) | Model 1 (1985-2000)  Age(ref: <20 years)  40+ yrs - S  0.45(0.42-0.49)  30-39 yrs - S  0.55(0.53-0.58)  20-29yrs - S  0.75(0.72-0.79)  Male (ref: female): - S  1.16(1.13-1.19)  Model 2 (2001-2006)  Age(ref: <20 years)  40+ yrs - S  0.55(0.49-0.61)  30-39 yrs - S  0.75(0.69-0.82)  20-29yrs - S  0.84(0.77-0.91)  Male (ref: female): - S  1.14(1.08-1.21) | - | Model 1 (1985-2000)Intake (ref:1985-1989)  1995-2000 – S  1.24(1.19-1.28)  1990-1994 – NS  0.96(0.92-1.00)  f**irst 9 months of treatment (ref: clinic)**  Correctional facility - S  0.61(0.56-0.65)  Community pharmacy - S 0.68(0.64-0.72)  Other - S  0.83(0.76-0.90)  **after 9 months in treatment (ref: clinic)**  Correctional facility - S  1.18 (1.09-1.28)  Community pharmacy - S 0.86(0.80-0.92)  Other – NS  1.09(.99-1.20)  Model 2 (2001-2006) Buprenorphine (ref Methadone) – S  1.89(1.79-1.99)  f**irst 6 months of treatment (ref: clinic)**  Correctional facility - S  0.76(0.69-0.83)  Community pharmacy - S 0.44(0.38-0.50)  Other - S  0.96(0.85-1.08)  **After 6 months in treatment (ref: clinic)**  Correctional facility - S  1.94 (1.76-2.15)  Community pharmacy - S 0.45 (0.56-0.76)  Other – NS  0.63 (0.49-0.82) | - | - | - | - |
| Cao 2014 (45) | Retention at 6 years (35.7%) | Dropout (follow-up time 6 years)  Cox proportional hazards  Hazard Ratios (95% CI) | - | Illicit drug use during treatment (ref: ≤ 10%):11-20% - S  0.55(0.42-0.73)  >20% - S  0.63(0.52-0.76) | Dosage (ref: ≤ 30%)  31-60mg – S  0.57(0.47-0.71)  >60mg – S  0.38(0.29-0.51) | - | - | Contact with drug users (ref: none)  1-30 – NS  0.93(0.76-1.45)  >30 – NS  1.14(0.90-1.45)  Relatives who were MMT clients (ref: no)  Yes – S  0.72(0.54-0.96) | - |
| Cox 2013 (46) | Retention at 12 months (72%)  Retention at 3 years (63%) | Dropout (time to dropout between 2004 and 2007)  Cox Proportional Hazards Regression models of (1) Involuntary discharge; (2) voluntary discharge  Hazard Ratios (95% CI) | Model 1: Involuntary Discharge  Sex (Female) – S  0.31 (0.13-0.71)  Model 2: Voluntary discharge  Sex (Female) – S  2.47 (1.11-5.54) | Model 1: Involuntary Discharge  Use of sedatives- S  3.56 (1.77-7.19)  Lifetime history of opioid abuse (> 5 years) - S  2.05 (1.06-3.98)  Model 2: Voluntary discharge  Injecting opioids – S  4.44 (1.64-11.99)  Use of sedatives- S  3.69 (1.61-8.47) | - | Injecting opioids – S  4.44 (1.64-11.99) | - | Model 1: Involuntary Discharge    Number of lifetime arrests >6 - S  2.70 (1.14-5.17)  Any personal debt – S  0.44( 0.21-0.91  Days of serious conflict with others (>- 1 day excluding family) - S  4.40 (2.11-9.21) | - |
| Cunningham 2013 (47) | Retention at 6 months (54%) | Retention at 6 months  Logistic regression  Odds Ratios (95% CI) | - | Prior use of illicit buprenorphine only - NS  2.92(0.95-8.91) | Prior use of prescribed buprenorphine – NS  2.53(0.81-7.88)  Prior use of any buprenorphine –S  2.65(1.05-6.70) | - | - | - | - |
| Davstad 2007 (48) | Retention at 1 year (84%); retention at 2 years (65%) | Dropout between 1995 and 2000  Poisson Regression and Spearman’s rank order correlations | - | - | Last mean adjusted dose was significantly lower for discharged patients than for those who remained in treatment:  70mg vs. 76mg at 6 months – S  p<.0001  75mg vs. 84mg at 1 year – S  p<.0001  81mg vs. 87mg at 2 years - S  p<.05 | - | - | - | - |
| Dayal 2017(49) | Retention at 6 months (19.1%); at 12 months (11.7%) | Dropout at 2 years  Cox Regression    Hazard Ratios (95% CI) | - | Benzodiazepine use (ref: no) - NS  0.58 (0.21-1.60) | Buprenorphine maintenance dose - S  0.86 (0.78-0.95) | Past month injection use (yes)– S  0.31(0.14-0.66) | - | Use of substance in 1st degree relatives (ref: no) – S  2.40 (1.34-4.31)  Parent support (ref: no) - NS  0.99(0.57-1.73) | - |
| Deck 2005 (50) | Retention  NR | Retention at 12 months  Logistic regression  Odds Ratios (95% CI) | **Oregon**  Age (in 10 year increments) – S  1.25(1.09-1.43)  Male – S  0.79(0.67-0.95)  African American (vs Caucasian) – NS  0.79(0.58-1.08)  Hispanic (vs. Caucasian) – NS  1.01(0.64-1.57)  Native American (vs. Caucasian) – NS  0.81(0.51-1.28)  **Washington**  Age (in 10 year increments) – S  1.21(1.06-1.38)  Male – S  0.73(0.61-.88)  African American (vs Caucasian) – S  0.49(0.38-0.64)  Hispanic (vs. Caucasian) – NS  0.96(0.67-1.40)  Native American (vs. Caucasian) – NS  0.70(0.45-1.09) | **Oregon**  Cocaine as secondary drug – S  0.69(0.62-0.88)  Daily opiate use in past 30 days – S  0.68(0.49-0.94)  Alcohol as secondary drug – NS  1.03(0.84-1.26)  Amphetamines as secondary drug – S  0.76(0.57-0.83)  Year of opiate use (log transformation – NS  1.09(0.95-1.25)  **Washington**  Cocaine as secondary drug - S  0.64(0.54-0.76)  Daily opiate use in past 30 days – S  0.78(0.61-0.99)  Alcohol as secondary drug – NS  1.00(0.83-1.20)  Amphetamines as secondary drug – NS  1.01(0.70-1.45)  year of opiate use (log transformation) – NS  1.08(0.95-1.22) | **Oregon**  Prior methadone (past 2 years) – S  1.44(1.17-1.79)  Prior treatment (past 2 year) – NS  1.17(0.97-1.41)  Self-referral – NS  0.86(0.67-1.12)  Stable admission cohort (vs 1994)  1995 - NS  1.23(0.95-1.58)  1996 – NS  1.29(0.95-1.75)  1997 – S  1.97(1.44-2.71)  1998 – S  3.54(2.61-4.80)  1999 – S  2.85(2.06-3.93)  Treatment agency referral – NS  1.05(0.78-1.39)  Legal referral - NS  1.10(0.56-2.15)  **Washington**  Prior methadone (past 2 years) – NS  1.23(.97-1.56)  Prior treatment (past 2 year) – NS  0.99(0.82-1.19)  Admission cohort (vs 1994)  1995 – NS  1.01(0.79-1.29)  1996 – NS  1.07(0.80-1.44)  1997 – S  1.40 (1.06-1.86)  1998 – NS  1.22 (0.90 -1.67)  1999 – NS  1.12(0.83-1.52)  Self-referral – NS  0.96(0.72-1.28)  Treatment agency referral – NS  1.48(0.92-2.40)  Legal referral – NS  1.24(0.75-2.02) | **Oregon**  Needle use in past 30 days – NS  0.84(0.61-1.16)  **Washington**  Needle use in past 30 days – NS  0.75(0.53-1.06) | **Oregon**  Disabled (vs. welfare) – S  0.60(0.43-0.84)  Expansion (vs welfare) – NS  0.80(0.61-1.05)  Other (vs welfare) – NS  0.75(0.52-1.07)  Mental health needs - NSU-  Stable medicaid eligibility - S  2.45(1.98-3.04)  **Washington**  Disabled (vs. welfare) – NS  1.16(0.92-1.48)  Expansion (vs welfare) – NSU-  Other (vs welfare) – NS  1.23(0.96-1.59)  Mental health needs - NS  1.05(0.87-1.26)  Stable medicaid eligibility - S  1.64(1.32-2.05) | **Oregon**  Married (vs other status – NS  1.10(0.88-1.37)  Never married (vs other status) – NS  1.08(0.89-1.30)  Not employable – NS  0.83(0.67-1.04)  No source of income – NS  0.90(0.74-1.10)  Personal home (vs other living situation) - NS 0.94(0.74-1.17)  Homeless(vs other living situation) – NS  0.99(0.70-1.41)  Pregnant – NS  0.72(0.44-1.20)  Arrested (past 2 years) – S  0.81(0.68-0.96)  **Washington**  Married (vs other status) – NS  1.26(0.99-1.59)  Never married (vs other status) – S  1.24(1.02-1.52)  Not employable – NS  0.89(0.71-1.11)  No source of income – NS  1.08 (0.80-1.45)  Personal home (vs other living situation) – NS  1.06(0.81-1.40) Homeless(vs other living situation) – NS  0.87(0.58-1.32)  Pregnant – S  0.42(0.26-0.67)  Arrested (past 2 years) – S  0.73(0.61-0.87)  Enrolled in Alcoholism and Drug Addiction Treatment and Support Act system – S 0.54 (0.33-0.89) | **Oregon**  Adjacent county (vs. county with clinic) – NS  1.11(0.86-1.44)  Distant country (vs county with clinic) – NS  0.71(0.41-1.24)  Agency provider (vs mean)  A – NS  0.97(0.79-1.20)  B – NS  0.95(0.79-1.14)  C – NS  0.99(0.78-1.25)  D – S  1.46(1.05-2.03)  E – NS  0.80(0.62-1.04)  F – S  0.42(0.33-0.54)  G – S  4.41(2.76-7.05)  **Washington**  Adjacent county (vs. county with clinic) – NS  0.99 (0.68-1.29)  Distant country (vs county with clinic) – NS  0.75(0.43-1.31)  Agency provider (vs mean)  A - NS  0.88(0.69-1.12)  B – NS  0.89(0.69-1.69)  C – NS  1.24(0.95-1.63)  D – NS  0.78(0.54-1.15)  E – S  5.94(2.71-12.99)  F – S  0.31(0.16-0.59) |
| Dumchev 2017 (51) | Retention  Retention at 6 months (75.8%); 9 months (70.1%); 12 months (65.8%); 18 months (59.4%); 24 months (53.8%);60 months (39.6%) | Dropout (time to dropout between 2005 and 2012)  Cox proportional hazards model  hazard ratio (95% CI) | Age – NS  1.00(1.00-1.00)  Sex (male vs female) – NS  1.00(0.94-1.07) | - | Medication type (buprenorphine vs methadone) – NS  1.00(0.94-1.06)  Last prescribed dose (high vs low) – S 0.57(0.44-0.75)  Last prescribed dose (medium vs low) – S 0.57(0.45-0.71)  Admission year:  2010 vs 2011-2012 – NS  1.00(0.95-1.05)  2009 vs 2011-2012 - NS  0.99(0.92-1.07)  2008 vs 2011-2012 - NS  1.00(0.96-1.05)  2007 vs 2011-2012 - NS  1.01(0.88-1.17)  2006 vs 2011-2012 - NS  1.00(0.94-1.06)  2005 vs 2011-2012 – S  3.39(2.04-5.63) | - | Last HIV test result (negative vs positive) – NS  1.00(0.96-1.04)  Last HIV test (not tested vs positive) – S  4.44(3.10-6.35)  Last HCV test result ( negative vs positive) - NS  1.00(0.92-1.08)  Last HCV test result (not tested vs positive) – NS  1.11(0.81-1.53)  Last HBV test results (negative vs positive) - NS  0.88(0.59-1.30)  Last HBV test (not tested vs positive) – NS  1.12(0.80-1.57)  Last TB test result (negative vs positive) – NS  0.78(0.51-1.18)  Last TB test result (not tested vs positive) – S  3.34(2.23-5.01) | - | Site (DPSPC vs DPGP5) –NS  1.17(0.74-1.85)  Site (PNDKR vs DPGP5) – NS  0.82(0.53-1.26)  Site (PNDN7 vs DPGP5) – NS  0.93(0.69-1.25)  Site (VOND1 vs DPGP5) – NS  1.23(0.78-1.92)  Site (VOND2 vs DPGP5) – NS  1.02(0.87-1.19) |
| Eibl 2015 (52) | Retention at 1 year  NR  (39.3%- 48.9%) | Retention at 1 year  Logistic regression  Odds ratios (95% CI) | Southern Rural (ref: Southern Urban) – NS  1.06(0.92-1.22)  Northern Urban – S  1.14(1.02-1.27)  Northern Rural – S  1.31(1.09-1.58) | - |  | - | - | - | - |
| Franklyn 2017(53) | Average retention rate at one year 43.4% | Dropout at 1 year  Cox proportional hazard model  Hazard Ratios (95% CI | Age - S0.98 (0.98–0.98)  Gender (ref: male) - S  0.77(0.70–0.84)  Location:  North (ref: south) – S  0.59(0.54–0.66)  Rural(ref: urban) - N S  0.98(0.86–1.12) | First-month benzodiazepine use – S  1.15(1.02–1.29) | - | - | - | - | - |
| Friedmann 2001 (54) | Retention in MMT at 1 year (49%) | Retention at 1 year  Multivariate logistic regression models  Odds Ratios (95% CI) | Age per 10 years - S  1.34(1.31-1.37)  Gender (ref: male):  female – NS  1.20(0.91-1.59)  Race (ref: Caucasian):  African American - S  0.69(0.49-0.97)  Other – NS  0.81(0.54-1.20) | Heavy alcohol use (ref: no):  Yes – NS  0.98(0.67-2.86)  Primary drug type (other drug: ref):  Heroin – NS  1.01(0.67-1.52)  Cocaine – NS  0.69(0.39-1.22)  Drug severity composite score – NS  1.06(0.38-2.93) | Number prior drug treatments – NS  1.01(0.98-1.04) | - | Psychiatric severity composite score - NS 0.70(0.27-1.85)  Medical severity composite score - NS 1.15(0.70-1.90)  Insurance type (ref: uninsured):  Private – NS  1.36(0.84-2.21)  Public - NS  1.10(0.79-1.55) | Any criminal status (ref: no):  Yes – NS  0.99(0.74-1.33)  Employment status (ref: no work):  Legal work – NS (0.87 0.62-1.22)  Illegal work – NS  0.74 (0.51-1.07) | Provision of transportation assistance (ref: no):  Yes – S  2.97(2.10-4.20)  Individual vouchers or payment (ref: no):  yes - NS  0.96(0.71-1.29)  Client capacity per 10 clients – NS 1.00(0.99-1.01)  Treatment readiness score – NS  1.06(0.60-1.88) |
| Gerra 2011(55) | Dropout at 12 months  Clinic A (42%); Clinic B (26%); clinic C (50%)  Retention at 12 months  60.66% | Dropout at 1 year  Event history analysis  Odds Ratios ( p values) |  |  | MMT Policy (Clinic A: under supervised daily consumption:  Clinic B (contingent take home)-S  0.535 (p=0.02)  Clinic C (non-contingent take home) –NS  1.144 (p=0.56) |  |  |  |  |
| Gryczynski 2014(56) | Treatment dropout at 6 months 42.1% | Time to dropout at 6 months  Cox proportional hazards model  Hazard Ratio (95% CI) | Female-NS  0.91 (0.63-1.32)  Age-S  0.95 (0.91-1.00[)  [time varying covariates separate model:  Age-S  1.0006 (1.0002-1.0012)] | Intensive outpatient counselling (ref standard counselling)-NS  0.96 (0.67-1.38)  Buprenorphine dose-S  0.84 (0.78-1.89)  [time varying covariates separate model:  Buprenorphine dose-S  1.002 (1.001-1.003)] | Injection drug users-NS  1.42 (0.94-2.13)  Baseline positive cocaine urine-S  1.71 (1.18-2.48) |  |  |  | Intended treatment duration (weeks)- NS  1.004 (0.97-1.04) |
| Gu 2012 (57) | 51.3% of participants had dropped out of MMT at completion | Dropout at 18 months  Cox Regression  Risk Ratio (95% CI) | - | - | - | - | - | - | MMT is intended primarily for detoxification (Agree) (appropriate perception = 1): misconception - NS 2.29(0.97-5.41)  After using MMT for 2-3 months one could be detoxified and can quit using MMT (Agree) (appropriate perception = 1): misconception – S  3.10(1.84-5.25)  MMT requires a long term or even life time treatment (disagree) (appropriate perception=1): misconception – S  2.22(1.12-4.38)  One should reduce the dosage of methadone as it is harmful to one’s health (agree)(appropriate response=1): misconception – S  2.07(1.05-4.07)  Number of items reflecting misconception (0-1=1):  2-3 – S  3.80(1.26-11.49)  4 – S  7.13(2.38-21.37) |
| Haddad 2013 (58) | 56.8% retained at 6 months | Dropout at 6 and 12 months  Cox proportional hazard  Model 1 (dropout at 6 months)  Model 2: Dropout at 12 months  Hazard Ratio (95% CI) | **Model 1**  Age-S  0.96 (0.94-0.98)  Female-S  0.59 (0.37-0.92) | **Model 1**  Baseline cocaine screen Positive-S  2.18 (1.35-3.50)  **Model 2**  Baseline cocaine screen Positive-S  3.12 (1.57-6.16) | **Model 1**  Receipt of substance abuse counselling-S  0.54(0.36-0.79)  **Model 2**  Receipt of substance abuse counselling-S  0.34(0.19-0.59) |  | **Model 1**  Prescribed psychiatric medication-NS  0.69 (0.47-1.01)  HCV Positive-S  0.56 (0.36-0.86)  **Model 2**  Prescribed psychiatric medication-S  0.36 (0.20-0.62) |  |  |
| Huissoud 2012 (59) | Retention in treatment at 1 year (69%); at 3 years (45%) | Dropout (time to dropout between 2001 and 2008)  Multivariate Cox regression model  Hazard Ratios (p values) | Younger than 30 years – S  1.29(p = .001)  Gender – NS  (no figure reported) | - | Methadone maintenance dosage reached at the beginning of treatment – NS (no figure reported)  Participants in a first treatment – S  1.31 (p = .001)  Methadone delivered (4 times a week) – NS  p=0.52 | - | State of health as estimated by the doctor – NS  (no figure reported) | No fixed abode or living in an institution/prison – S  1.82 (p=.001) | Interaction between the mode of delivery and living condition – NS  (p = 0.13)  Source of income – NS  (no figure reported) |
| Johns 2018 (60) | Retention at 12 months (85%) | Dropout at 1 year  Cox regression analyses  Hazard Ratios (95% CI) | **Model 1:**  Age (years) (ref: 30 – 40 years)  <30 years – NS  1.58 ( 0.68 – 3.65)  >40 years – NS  0.74 ( 0.28 – 1.99)  Gender (percentage female) (ref: male) – NS  3.05 ( 0.41 – 22.50) |  | **Model 1:**  Length of time on MMT before entering study:  (ref: > 12 months to 24 months)  One month or less – NS  0.18 (0.03 – 1.12)  Less than one month to three months – NS  0.53 (0.17 – 1.67)  Less than 3 months to 12 months – NS  0.93 ( 0.43 – 2.02)  Methadone dose at three months of MMT (mg) (ref: 60 or more)  Less than 60mg – NS  1.36 ( 0.56 – 3.29)  Missing – NS  0.47 ( 0.10 – 2.12) |  | **Model 1:**  HIV status at baseline (self-reported): positive (ref: no) – S  0.24 ( 0.09 – 0.66)  ) | **Model 1:**  Marital status at baseline (ref: widowed/divorced/separated)  Single – NS  0.79 (0.30 – 2.12)  Married/long-term partner – S  0.29 (0.10 – 0.80)  Highest education achieved at baseline (ref: secondary/high school or more)  Less than secondary school – NS  1.26 ( 0.51 – 3.12)  Employment at baseline (ref: full-time, part-time, or self-employment)  Homemaker, retired or student – S  2.78 (1.54- 4.99)  Not working (seeking or not) – NS  0.49 ( 0.16 – 1.45)  Change of employment status in year before start of study (ref: no) – NS  0.83 ( 0.41 – 1.68)  Size of household (baseline) – S  0.83 ( 0.07 – 0.97) | **Model 1:**  Travel more than 30 minutes (one way) to MMT clinic (ref: no)  Yes - NS  1.50 ( 0.51 – 4.42)  Amount paid for transport to MMT clinic at baseline (VND thousands) – 1.04 (0.69 – 1.57)  Household expenditures at baseline (annual VND thousands) – S  0.95 (0.91 – 0.99)  Income below poverty line (ref: no) – NS  1.17 ( 0.48 – 2.84)  Clinical province had user fee policy (ref: no) – NS  0.17 ( 0.02 – 1.29) |
| Kayman 2006 (61) | Retention at 1 year (52%) | Dropout at 1 year  Logistic regression  Odds Ratios (95% CI) | Mean age – NSU  Female – NSU  Ethnicity (white and other, African American, Hispanic) – NSU | Cocaine use in past 30 days – NSU-Alcohol use in past 30 days – NSU- | Been in MMT program – NR  Ever in drug detoxification (0=no, 1=yes) – S  1.9 p=.00 | Injecting in the past 30 days – NSU- | - | Never married – NSU-  Lacks high school diploma – NSU- | Opinions about methadone (1-5 low score = less favourable opinions) – S  0.90 p=0.01 |
| Kelly 2011 (62) | Retention at 12 months (58.4%) | Dropout at 1 year (starting at 3 months)  Cox proportional hazards regression  Hazard Ratios (95% CI) | Age – NS  0.98(0.95-1.02)  Gender – NS  1.50(0.84-2.70)  Race – NS  1.21(0.56-2.60) | **Baseline variables**  Smoked crack/cocaine (lifetime) – NS  1.05(0.62-1.77)  Alcohol composite – NS  0.24(0.03-1.95)  Drug composite – NS  2.90(0.15-56.82) | **3 month variables**  Dose – S 0.99 (0.98-1.00)  Counsellor – NS 1.00 (.97 – 1.04) | - | **Baseline variables**  Psychiatric composite (self-reported)– NS  0.72(0.13-3.96)  Medical composite (self-reported) –S  0.31(0.10-0.93) | **Baseline variables**  Employment composite – NS  2.43(0.68-8.65)  Family/social composite – NS  3.45(0.56-21.38)  Legal composite – S  3.60 (1.03-12.63)  On parole or probation – NS  1.12(0.63-1.97) | **Baseline variables**  Problem recognition – NS  0.97(0.92-1.03)  Desire for help – NS  0.99(0.89-1.11)  Treatment readiness –NS  1.08(0.99-1.18)  **3 month variables**  Treatment satisfaction – S  0.91(0.84-0.99)  Problem recognition – NS  1.04(0.99-1.09)  Desire for help – NS  0.94(0.85-1.03)  Treatment readiness –N S  0.98(0.91-1.04) |
| Lambdin 2014 (63) | Retention at 6 months (67%); 12 months (57%); and 24 months (48%) | Dropout (time to dropout between 2011 and 2013)  Cox proportional hazards  Hazard Ratios (95% CI) | Age (ref: ≤ 25):  26-35 - S  0.35(0.23-0.52)  36-45 – S  0.25(0.16-0.39)  >45 – S  0.11(0.03-0.38)  Gender (female) 0.50 (0.28-0.90) | - | Methadone dose at initiation (ref:<40mg)  40-85mg - S  0.50(0.37-0.68)  >85mg – S  0.41(0.29-0.59) | Risky sexual behaviour in the last 6 months – NS  1.29(0.99-1.68) | Any history of sexual abuse – S  2.84(1.24-6.51) | - | - |
| Ledgerwood 2019 (64) | NR | Retention (days retained between 2002 and 2009)  Multiple Linear regression analyses  Beta (P values) | African American – NS  0.07 (0.398)  ≥ high school education – NS  -0.05 (0.446) | Early opioid use onset ( < 21 years) - NS  -0.06 (0.334)  Past year cocaine use disorder – S  -0.26 (<0.001) |  | Injection use – NS  -0.06 (0.371) | Hepatitis C positive – NS  0.01 (0.927) |  |  |
| Lin 2013(65) | Retention at 6 months (61.5%); 12 months (41.6%; 18 months (32.3%) | Dropout at 18 months  (from 3 months)  Cox proportional hazards model  Hazard Ratios (95% CI) | Model 1:  Age – NS  0.99 (0.96–1.01)  Male – NS  1.34 (0.87–2.16)  Location - NS  Model 2:  Age – NS  1.00(0.98-1.04)  Male – NS  1.33(0.72-2.66)  Location - NS  Race - NS | Model 1:  Severity of heroin dependence on the Chinese Version of the Severity of Dependence Scale (SDS) – NS  0.96 (0.91–1.01)  Harmful impact of heroin use on the Questionnaire for the Harm of Opioid Use (Q-HOU) - S  1.04 (1.00–1.07)  Lifetime methamphetamine use – NS  1.01 (0.75–1.37)  Age of initial heroin use – NS  1.02 (0.99–1.05)  Model 2:  Severity of heroin dependence on the Chinese Version of the Severity of Dependence Scale (SDS) – NS  0.95(0.87-1.02)  Harmful impact of heroin use on the Questionnaire for the Harm of Opioid Use (Q-HOU) - S  1.05(1.00-1.11)  Lifetime methamphetamine use – NS  0.98(0.65-1.50)  Age of initial heroin use – NS  1.00(0.97-1.04) | Model 1:  Being compulsory to receive MMT by the court – NS  1.34 (0.86–2.18)  Model 2:  Methadone dose at 3 months after starting MMT - S  0.99(0.98-1.00)  Being compulsory to receive MMT by the court – NS  1.26(0.69-2.25) | - | Model 1:  Clinical diagnosis:  Severity of depression on the Center for Epidemiological Studies –Depression Scale (CES-D) – NS  1.01 (0.99–1.02)  Positive serum HIV antibody test – NS  0.76 (0.47–1.19)  TB +/- ns  Model 2:  Clinical diagnosis:  Severity of depression on the Center for Epidemiological Studies –Depression Scale (CES-D) – NS  1.00(0.98-1.02)  Positive serum HIV antibody test – NS  0.96(0.54-1.62)  TB +/- ns | Model 1:  Unmarried or divorced – NS  0.87 (0.60–1.30)  Regular employment - NS  0.98 (0.74–1.30)  Education level less than or equal to 9 years - NS  0.87 (0.65–1.16)  Family support on the Family APGAR index – S  0.96 (0.93–1.00  Prior criminal records – NS  0.99 (0.73–1.34)  Model 2:  Unmarried or divorced – NS  0.72(0.43-1.27)  Regular employment - NS  0.84(0.58-1.23)  Education level less than or equal to 9 years - NS  0.95(0.65-1.40)  Family support on the Family APGAR index – NS  0.97(0.92-1.02)  Prior criminal records – NS  1.23(0.81-1.85) | Model 1:  High monthly cost of heroin – S  1.43 (1.07–1.91)  High economic burden related to heroin use – NS  1.1 (0.81–1.49)  Advantages of heroin use on the Decision Balance Scale (DBS) – NS  1.00 (0.96–1.04)  Disadvantages of heroin use on the DBS - NS  0.99 (0.96–1.02)  Travel time to MMT – NS  1.00 (0.99–1.01)  Acceptable weekly expense for receiving MMT – NS  1.00(1.00-1.00)  Acceptable daily time spent receiving MMT – NS  1.10 (0.94–1.27)  Unfavourable attitudes towards MMT on the Client Attitudes toward Methadone Programs Scale (CAMP)- NS  0.97 (0.90–1.05)  Pre-contemplation stage on the Readiness to Change Questionnaire (RCQ-12) – NS  1.02 (0.95–1.09)  Contemplation stage on the RCQ-12- NS  1.02 (0.96–1.07)  Action stage on the RCQ-12 – NS  1.00 (0.93–1.08)  Model 2:  High monthly cost of heroin – NS  1.27(0.86-1.87)  High economic burden related to heroin use – NS  1.10(0.74-1.66)  Advantages of heroin use on the Decision Balance Scale (DBS) – NS  1.03 (0.97-1.08)  Disadvantages of heroin use on the DBS - NS  1.01(0.97-1.06)  Travel time to MMT – NS  0.99(0.98-1.00)  Acceptable weekly expense for receiving MMT – NS  1.00(1.00-1.00)  Acceptable daily time spent receiving MMT – NS  1.09(0.87-1.33)  Unfavourable attitudes towards MMT on the Client Attitudes toward Methadone Programs Scale (CAMP)- NS  0.99(0.89-1.10)  Pre-contemplation stage on the Readiness to Change Questionnaire (RCQ-12) – NS  1.03(0.94-1.13)  Contemplation stage on the RCQ-12- NS  0.98(0.91-1.06)  Action stage on the RCQ-12 – NS  0.96(0.87-1.07) |
| Lin 2015 (66) | Retention at 6 months (73.3%); 12 months (61.1%); at 18 months (48.9%) | Dropout at 18 months  Cox multivariate regression  Hazard Ratios (95% CI) | Age – NS  1.01 (.98-1.04)  Age at first heroin use – NS  0.99(0.95-1.04)  Gender (male) - S  0.45(0.21-0.96) |  | methadone dose after 30 days (mg) S  0.99 – S (0.98 - 0.99),  Program site (new Tapei city) - S  2.84 (1.06 -7.58)  Program site (Keelung city) – NS  0.59(0.23 - 1.52)  program site(Yilan city) – NS  2.8(0.96-8.73) |  | HIV sero-status (positive) - NS  0.55 (0.25 - 1.22)  HCV stero-status (positive) - S  3.91 (1.01-15.12) | Incarcerated during study period (no) – S  0.44 (0.25 - 0.76)  Education (at least 9 years) – NS  1.05 (0.61 - 1.79)  Employed (yes) - NS  0.76(0.40 - 1.44)  Marital status (single) - NS 1.28 (0.50 -3.30)  Marital status(divorce/widowed)- NS 1.79(0.89 - 3.60) | House to clinic distance(km) –  S  1.08 (1.04-1.13) |
| Liu 2017 (67) | Dropout within 12 months (21%) | Dropout at 12 months  Cox regression model  Hazard Ratios  (95% CI) | - | Over-drinking alcohol (ref: no) - NS  1.17(0.72-1.92)  Drinking frequency (ref: never) at least once – NS  0.84(0.54-1.29)  Drinks on a typical day when drinking (ref: 1-2 drinks):  > 3 drinks – NS  1.38(0.71-2.69)  Frequency of having 6 drinks or more on one occasion when drinking (ref: never) at least once – NS  1.94(1.00-3.78)  Use of methamphetamine in the last 6 months (ref: no) – S  2.26(1.15-4.43)  Use of MaGU ( methamphetamine and caffeine) in last six months (ref: no) – NS  1.79(0.55-5.80)  Use of ketamine in the last six months (ref: no) – NS0.91(0.12-6.78)  Use of ecstasy in the last six months (ref: no) – NS  1.63(0.22-12.23)  Use of any club drugs in the last 6 months (ref: no) –S  1.90(1.01-3.56)  Age of first drug use – NSU  Use of triazolam in the last 6 months (ref: no) –NS  1.51(0.21-10.97) | No. of times compulsory drug detoxification  three times or more versus never – S0.36(0.16–0.83) | - | - | Current marital status: divorced or other (ref: single) – S  2.10(1.13-3.89)  (data not tabulated) | - |
| Manhapra 2017 (68) | Retention > 1 year (61.6%); retention for > 3 years (31.8%) | Dropout (follow-up 3 to 4 years)  Cox proportional hazard analysis  Hazard Ratios (p values) | Black race – S  1.26 ( p=0.003 |  |  |  | Charlson comorbidity index – S  1.03 p=0.013) (  Visits to the emergency room during FY 2012 – S  1.03 p<0.0001 |  |  |
| Manhapra 2018 (69) | 45% retained in treatment for more than one year; 13.7% retained at > 3 years | Dropout (follow-up 3 years)  Cox proportional hazards model  Hazard Ratios (95% CI) | Above median age of 31 years – S  0.82 ( 0.80 – 0.85)  **Model 2:**  Above median age of 31 years – S  0.90( 0.87 – 0.93) | Any substance use under disorder diagnosis – S  1.06 ( 1.02 – 1.10)  **Model 2:**  Any substance use under disorder diagnosis – S  1.05 ( 1.01 – 1.10) |  |  | Inpatient mental health treatment in fiscal year (FY) 2011 – S  1.20 ( 1.19 – 1.30)  Emergency department visits in FY 2011 - S  1.10 (1.06 – 1.14)  Any psychiatric diagnosis – S  1.05 (1.01 – 1.09)  Any psychotherapy in FY 2011 – S  0.86 ( 0.83 – 0.89)  **Model 2:**  Inpatient mental health treatment in fiscal year (FY) 2011 – S  1.30 ( 1.24 – 1.36)  Emergency department visits in FY 2011 - S  1.07 (1.04 – 1.14)  Any psychiatric diagnosis – S  1.05 (1.01 – 1.10)  Any psychotherapy in FY 2011 – S  0.90 ( 0.86 – 0.92) |  | **Model 2:**  Each 30 days of insurance enrolment after buprenorphine initiation – S  0.90( 0.90 – 0.91) |
| Meshberg – Cohen 2018 (70) | Retention at 6 months (64%) | Retention at 6 months  Logistic regression model  Odds Ratios  (95% CI | Age – S  1.04 ( 1.01 -1.08) | Heroin use – S  0.26 ( 0.11 – 0.64) |  |  | PTSD diagnosis + PTSD treatment – S  43.36 (8.10 – 232.06)  No PTSD diagnosis – NS  2.20 (0.86 – 5.70) |  | Service connection status – NS  0.95 (0.39 – 2.29) |
| Monico 2015 (71) | Retention  NR | Retention at 6 months  Logistic regression  Odds Ratios (95% CI) | Age (in years) – NS  1.00(0.97-1.04)  Gender (ref: female) – NS  0.93(0.57-1.52) | Co-occurring cocaine use (ref: no) – S  0.48(0.29-0.80)  Prior illicit buprenorphine Use (ref: no) – NS  2.09(1.23-3.56) | Prior buprenorphine treatment (ref: no) – NS  1.03(0.62-1.69)  Clinic site (ref: site 1) – NS  0.97(0.59-1.61) | - | - | - | - |
| Montalvo 2019 (72) | Retention at one year (52.6%); at 2 years (35.5%) | Retention at 1 and 2 years  Model 1: Multiple logistic regression of retention at 1 year  Model 2: Multiple logistic regression of retention at 2 years  Odds Ratios  (95% CI) | **Model 1:**  Age – NS   - 1. (0.98 – 1.04)   Gender (ref: male) – NS  0.95 (0.54 – 1.67)  Ethnicity (ref: white)  Black/African-American – NS  1.71 ( 0.67 – 4.39)  Hispanic/Latino – NS  0.51 (0.12 – 2.17)  Denied/not available – NS  0.81 (0.31 – 2.09)  Other – NS  2.73 (0.08 – 88.46)  **Model 2:**  Age – NS   - 1. (0.99 – 1.05)   Gender – S  2.08 (1.16 – 3.73)  Ethnicity (ref: white)  Black/African-American – NS  2.28 ( 0.92 – 5.64)  Hispanic/Latino – NS  0.78 (0.15 – 4.17)  Denied/not available – NS  1.56 (0.59 – 4.12)  Other – NS  11.55 ( 0.24 – 551.23) | **Model 1:**  Stimulant use disorder – NS  1.13 (0.56 – 2.31)  Alcohol use disorder – NS  1.36 ( 0.63 – 2.92)  Nicotine use – S  2.40 (1.35 – 4.27)  Other substance use order – NS  1.51 (0.86 – 2.67)  **Model 2:**  Stimulant use disorder – NS  0.57 (0.28 – 1.18)  Alcohol use disorder – NS  1.18 ( 0.56 – 2.49)  Nicotine use – S  2.00 (1.13 – 3.52)  Other substance use order – NS  1.46 (0.79 – 2.70) |  |  | **Model 1:**  Depressive order – NS  2.16 (0.99 – 4.70)  Other mood disorder – S  3.42 ( 1.95 – 5.98)  PTSD – NS   - 1. ( 0.56 – 1.89)   Co-prescription of medications with buprenorphine:  Benzodiazepines – S  2.44 (1.30 – 4.56)  Clonidine – NS  1.29 ( 0.64 – 2.60)  Gabapentin – NS  1.42 ( 0.81 – 2.47)  Stimulants – NS  1.31 (0.67 – 2.54)  **Model 2:**  Depressive order –S  4.61 (1.49 – 14.29)  Other mood disorder – S  3.60 ( 1.88 – 6.88)  PTSD – NS  1.28 ( 0.70 – 2.35)  Co-prescription of medications with buprenorphine:  Benzodiazepines – NS  1.74 (0.94 – 3.25)  Clonidine – NS  1.18 ( 0.58 – 2.37)  Gabapentin – NS  1.15 ( 0.64 – 2.06)  Stimulants – NS  1.34 (0.68 – 2.64) |  |  |
| Mullen 2012 (73) | Retention at 12 months (60.7%) | Retention at 12 months  Logistic regression    Odds Ratios (95% CI) | Gender (ref: female) – S  1.40(1.00-2.00)  Age -NSU | - | Facility type (ref: primary care setting):  Specialist treatment centre – S  2.00(1.20-3.50)  Community treatment centre – NS  1.20(0.80-1.70)  Average methadone dose (ref: 60mg or more):  59mg or less – S  3.0(2.2-4.0) | Injecting drug use - NSU- | - | - | - |
| Nosyk 2009 (74) | Retention at 6 months (55.5%); at 12 months (42%); at 24 months (29.5%) | Proportional hazards frailty models to assess time to discontinuation from recurrent MMT episodes  Hazard Ratio (95% CI) | Gender (ref male) –NS  0.987 (0.955-1.019)  Age at treatment entry (ref <40 yrs)- S  0.872 (0.842-0.902) |  | Mean daily dose methadone (ref 40-<60)  <40 mg-S  1.207 (1.161-1.255)  60-<80-S  0.70 (0.674-0.729)  80-<100- S  0.539 (0.514-0.564)  100-<120-S  0.441 (0.414-0.469)  >120-S  0.377 (0.351-0.405)  Episode Number (ref. 1)  2- S  0.871 (0.843-0.901)  3-S  0.839 (0.803-0.878)  4-S  0.801 (0.754-0.850)  5-S  0.833 (0.77-0.902)  >6-S  0.791 (0.732-0.856)  Adherence (ref >90%)  70-90%- S  2.289 (2.213-2.366)  <70%-S  6.835 (6.534-7.150) |  | Clark Chronic disease score (ref 1)  2- S  0.927(0.890-0.965)  3-S  0.901( 0.863-0.940)  4-S  0.904 (0.865-0.944) | Education –S  1.004 (1.002-1.005)  Employment-NS  0.998 (0.994-1.001)  Income-S  1.003 (1.001-1.005) | Quartile of prescriber patient load (ref 1)  2- NS  0.976(0.938-1.015)  3-S  0.935 ( 0.898-0.974)  4-S  1.074 (1.030-1.121)  Calendar year – S  0.98 (0.97-0.99) |
| Peles 2008 (75) | Retention at 6 months:  Tel Aviv (83.5%); Las Vegas (78.5%)  Retention at 1 year:  Tel Aviv (74.4%); Las Vegas (61.6%) | Retention at 1 year  Logistic regression  Odds Ratios (95% CI)  Dropout (follow up 1-10 y in Tel-Aviv 1-4 y in Las Vegas)  Cox PH model  HR (“OR”) | **Tel-Aviv Clinic**  Older age – S  1.80(1.10-2.90)  Cox model  **Tel-Aviv Clinic**  Age – NS NR  **Las Vegas Clinic**  Age 30 years or more on admission – S  2.2 (1.4–3.6) | **Las Vegas Clinic**  No amphetamines on admission – S2.10(1.05-4.02)  **Las Vegas Clinic**  Amphetamines and cannabis use on admission – NS NR | Cox model  **Tel-Aviv Clinic**  Methadone dosage ≥ 100 mg/day after 1 year – S  2.1 (1.6–2.9)  **Las Vegas Clinic**  Methadone dosage ≥ 100 mg/day after 1 year –S  1.8(1.3–2.5) | Cox model  **Tel-Aviv Clinic**  No urine illicit opiates after 1 year – S  2.3 (1.7–3.1)  No urine illicit benzodiazepines after 1 year – S  1.4 (1.0–2.0)  **Las Vegas Clinic**  No urine cocaine after 1 year – S  1.4 (1.0–1.9)  No urine amphetamines after 1 year – S  1.7 (1.1–2.7) | **Las Vegas Clinic**  Sera-positive HCV – S  2.40(1.10-5.30)  TB +/- - N/A | **Las Vegas Clinic**  Having Children – S  1.90(1.10-3.30) | - |
| Peles 2018 (76) | Retention  Overall length of retention in treatment – 7.9 years (95% CI 7.4 – 8.5) | Retention at 24 years  Cox multivariate analyses  Hazard Ratios (95% CI) | Age ≥ 30 years (vs. < 30 years) – S  1.4 (1.1 – 1.7) | No opiate abuse after 1 year (vs. opiate abuse) – S  1.8 ( 1.5 – 2.1)  No benzodiazepine abuse after 1 year (vs. benzodiazepine abuse) – S  1.7 ( 1.4 – 2.1) | Methadone ≥ 100mg/day (vs. ≤ 100mg/day) – S  1.8 (1.5 – 2.2) |  | Axis I psychiatric diagnosis or none (vs. Axis II only) – S  1.9 ( 1.6 – 2.3)  Not admitted from hospital (vs. admitted from hospital) – S  1.6 (1.2 – 2.0) |  |  |
| Perreault 2005 (78) | Retention at 6 months (72%) | Dropout at 6 months  Cox proportional hazards model  Hazard Ratios (95% CI) | Age - NSU-  Gender (female) – NS  1.69 (0.79-3.60) | >8 days of cocaine consumption in 30 days before treatment -  S  2.17(1.02-4.64)Cocaine dependence – NS  1.12(0.52-2.40)  Duration of heroin consumption – NSU-  Daily usage of heroin 30 days before treatment (yes/no) - NSU-  Daily usage of heroin 30 days before treatment (in days) – NSU- | - | Working in the sex trade – NS  1.70(0.85-3.35)  Use of unclean needles – NSU- | - | No stable income – NS  2.07(0.65-6.54)  Homelessness – NSU-  Legal problems – NSU- | - |
| Perreault 2015 (77) | Retention at one year (78%) | Retention at one year  Logistic regression  Odds Ratios (95% CI) | Age – NS  1.01(0.94-1.09)  Male – NS  0.93(0.26-3.38) | - | Methadone dose – S  1.04(1.01-1.07) | - | Clinical diagnosis:  Psychological distress – NS  1.05(0.97-1.13) | Number of criminal charges – S  0.97(0.96-0.99) | Stages of change:  Contemplation – NS  1.10(0.33-3.64)  Self-esteem – S  0.85(0.74-0.97) |
| Proctor 2015 (79) | Retention at 6 months (46.8%); at 12 months (20.3%) | Dropout (treatment discharge) at 6 and 12 months  Logistic regression  Model 1( 6 months);  Model 2 (12 Months)  Odds Ratios (95% CI) | **Model 1: 6 month**  Ethnicity - NR  **Model 2: 12 months**  Age- NR  Gender- NR | **Model 1: 6 month**  Intake cannabinoids - NS  0.91(0.67-1.23)  Intake benzodiazepine - NS  1.33(0.98-1.80)  Intake cocaine - S  1.79(1.18-2.72)  Intake amphetamines - NS  1.57(0.92-2.69)  **Model 2: 12 months**  Intake cannabinoids - NS  1.39(0.73-2.65)  6 month cannabinoids - NS  1.78(0.78-2.64)  Intake benzodiazepine - NS  1.18(0.66-2.11)  6 month Benzodiazepines - NS  1.19(0.55-2.58)  Intake cocaine - S  3.71(1.35-10.17)  6 month cocaine UDS+ - NS  1.27(0.39-4.18)  Intake amphetamines NS  1.61 (0.50-5.24)  6 month amphetamines - NS  2.91(0.84-10.12)  6 months opioids S 2.13 (1.10-4.12) | **Model 1: 6 month**  Average daily methadone dosage - NR  **Model 2: 12 months**  Average daily methadone dosage - NR | - | - | **Model 1: 6 month**  Employment status- NR  Marital status- NR  **Model 2: 12 months**  Method of payment - NR | - |
| Ren 2013 (80) |  | Dropout (follow-up 3 to 5 years)  Cox proportional hazards regression  Hazard Ratios (95% CI) | Age (ref: greater than or equal to 50 years):  <30 – S  1.41(1.16-1.71)  30-39 - S  1.37(1.15-1.63)  40-49 - NS  1.15(0.98-1.38) | Urine test (ref: opiate-negative):  positive – S  1.69(1.51-1.89) | Average methadone dose at stable stage (ref: >65mg/day):  ≤35mg – S  1.39(1.19-1.63)  36-50mg – S  1.36(1.18-1.56)  51-65mg - S  1.20(1.03-1.39)  Methadone dose change over time (ref: stable): tapering – NS  1.07(0.90-1.27)  Increasing – S  0.57(0.49-0.67)  Unknown - S  12.77 (11.09-14.71) | Needle sharing (ref: no): yes – S1.29(1.06-1.58) | - | Education (ref: senior high school and above):  Elementary and below – S  1.48(1.17-1.87)  Junior high school – NS  1.00(0.90-1.10) | - |
| Ruadze 2016 (81) | Retention at 6 months (86%); 9 months (85%); 12 months (83%) | Retention at 9 and 12 months  Logistic regression of retention at 9 months (model 1); retention at 12 months (model 2)  Odds Ratios (95% CI) | **Retention at 9 months:**  Age (>40 vs. ≤ 40 years) - S  2.33(1.53-3.55)  Age (cont. variable) - S 1.06(1.05-1.09)  Gender (male vs. female) – S  3.18(.1.26-8.00)  **Retention at 12 months:**  Age (>40 vs. <= 40 years) – S  2.37(1.55-3.64)  Age (cont. variable) - S 1.06(1.04-1.10)  Gender (male vs. female) – NS  2.48(0.95-6.48) | - | - | - | - | - | - |
| Saloner 2017 (82) | Retained in treatment for at least 6 months (41%) | Retention at 6 months  Logistic Regression  Odds ratio (95% CI) | Age (ref: 18-34):  35-49 yrs – S  1.16(1.09-1.23)  >50 yrs – NS  1.04(0.96-1.13)  Female – S  0.88(0.84-0.93)  State of pharmacy (ref: Arizona) (used for location of patient)  California – NS  1.47(0.93-2.32)  Florida – NS  1.31(0.82-2.08)  Georgia – S  1.77(1.12-2.79)  Louisiana – S  1.94(1.23-3.08)  Maryland – S  2.48(1.57-3.9)  New jersey – S  2.37(1.44-3.91)  New York – S  2.79(1.8-4.33)  Pennsylvania – S  2.2(1.41-3.43)  Texas – S  1.81(1.15-2.84)  Washington – S  2.17(1.38-3.39)  Other states – S  1.86(1.19-2.9)  Non-minority population (standardised) - NS1.10(1.04-1.16) | - | Majority payer (ref: cash)  Medicaid fee-for-service – S  0.35 (0.31-0.39)  Medicare part D – S  0.33(0.30–0.37)  Third-party commercial – S  0.41(0.39-0.44)  Majority prescriber (ref: Primary care physician):  Psychiatrist – NS  1.00(0.92-1.10)  Other provider - S 0.73(0.68-0.79) | - | - | County median income (standardised) – NS  1.02(0.98-1.07) | Metropolitan status (ref: non-metro)  Large (>1 million people) – NS  0.90(0.79-1.03)  Medium (>250k-1 million) – NS  0.95(0.83-1.09)  Small (100-250k) - NS  1.1(0.95-1.27)  County opioid overdose death rate (ref: >18.1 per 100K):  <10 deaths per 100k – NS  0.93(0.83-1.03)  10.1-14 deaths per 100k – NS  0.93(0.83-1.04)  14.1-17.9 deaths per 100k – NS  1.01(0.91-1.12)  Crossed county lines for treatment – S0.92(0.87-0.97)  Primary care physician to population ratio (standardized) – NS  1.03(0.98-1.08)  DEA waivered ratio (standardized) – NS  1.00(0.96-1.04) |
| Sarasvita 2012 (83) | Retention at 6 months (61.3%) | Dropout (follow-up 6 months)  Cox proportional hazards model  Hazard Ratios (95% CI) | Age – S  0.91(0.85-0.97) | Lifetime heroin use – NSU- | Dose – S  0.49(0.28-0.84)  Take Home Dose (THD) – S  0.91(0.85-0.97)Previous  Clinical experience – NS  0.60(.32-1.13)  Interaction between clinic and experience and THD – S  1.07(1.00-1.15)  Perceived accessibility - S 0.95(0.92-0.98)  Expense of methadone treatment – NSU-  Treatment need - NSU-  Pressure for treatment – NSU-  Self-efficacy - NSU-  Treatment participation – NSU- | - | - | Perceived peer support – S  1.10(1.02- 1.19)  Family support - NSU-  Imprisonment – NSU- | Belief to the program – S  0.93(0.87-0.98) |
| Schuman-Olivier 2013 (84) | Retention at 12 months (40%) | Retention at 12 months  Logistic regression  Odds ratios (95% CI) | age - NR | No difference in 12 month retention based on past-year BZD misuse; BZD prescription; or combination (OR not reported) | - | - | - | - | - |
| Shakira 2017 (85) | 60.1% retention at 1 year | Dropout at 1 year  Cox proportional regression  Hazard Ratios (95% CI) | Sex -NSU | Lifetime on illicit drugs – NSU  Relapse on illicit drugs during MMT - NSU | Level of maintenance dose (ref: low dose <30 mg)  Intermediate dose 30-59mg – NS  0.55 ( 0.25 – 1.21)  High dose > 60 mg – S  0.24 ( 0.09 – 0.63)  Take away dose (ref: no) – S  0.18 ( 0.11 – 0.30) |  |  | Employment status (ref: none)  Part time – NS  0.68 (0.39 – 1.20)  Full time – S  0.50 ( 0.27 – 0.94) |  |
| Shcherbakova 2018 (86) | Retention at 6 months (53.6%); at one year (40.4%) | Retention at 1 year  Logistic regression analysis  Odds Ratios  (95% CI) | Age – S  1.04 (1.01 – 1.06)  Male – NS  0.83 ( 0.44 – 1.58) | Non – opioid substance use disorder – NS  0.85 ( 0.44 – 1.63)  Addiction treatment specialist (ref: yes) – S  0.40 (0.21 – 0.76)  Pre-index exposure to prescription opioids – NS  1.44 (0.78 – 2.63) | Year of therapy initiation (ref: 2013):  2010 – NS  1.89 (0.69 – 5.14)  2011 – NS  1.39 (0.57 – 3.39)  2012 – NS  0.98 ( 0.49 – 1.98)  2014 – NS  1.09 (0.46 – 2.60) |  | Charlson Comorbidity Index ≥ 1 – NS  0.73 ( 0.35 – 1.54)  Mental health disorder – NS  1.36 (0.66 – 2.80)  Concomitant antidepressants – NS  1.04 (.056 – 1.94)  Concomitant benzodiazepines – NS  1.17 (0.57 – 2.41)  Concomitant clonidine – NS  0.78 (0.36 – 1.69)  Concomitant prescription opioids – S  0.25 (0.12 – 0.51)  Concomitant anticonvulsants – NS  0.81 (0.35 – 1.89)  Concomitant antipsychotics – NS  1.05 (0.36 – 3.10)  Concomitant stimulants – NS  0.33 (0.11 – 1.01)  Concomitant hypnotics – NS  1.97 (0.42 – 9.28)  All-cause ER admissions – NS  0.59 (0.31 – 1.12)  All-cause inpatient hospitalisation – S  0.34 (0.12 – 0.76) |  | Insurance (ref: Medicaid) – S  0.33 ( 0.13 – 0.84) |
| Socias 2018 (87) | Retention at 6 months (52.6%); 12 months (38.5%); and 18 months (31.5%) | Retention at 6 months  General linear mixed effects analyses of association between ≥daily cannabis use and retention  Odds Ratios  (95% CI) | Age (per year older) – S  1.05 (1.04 – 1.06)  Gender (male) – NSU  Ethnicity (Caucasian) – NSU | ≥ Cannabis use – S  1.21 (1.04 – 1.41)  ≥ daily heroin injection – S  0.25 (0.22 – 0.29)  ≥ Daily prescription opioid use – S  0.37 (0.29 – 0.47)  ≥ Daily cocaine injection – NSU  ≥ Daily crack use – NSU  Heavy alcohol use – S  1.13 (1.00 – 1.26) | Calendar year of OAT initiation – NSU |  | HIV positive - NSU | ≥ High school education – NSU  Homeless – NSU  Incarceration – NSU |  |
| Stein 2005 (88) | Retention at 6 months (58%) | Dropout at 6 months  Log-rank test | Race (Caucasian) – NS  P=.095  Age –NS  Gender – NS | Cannabis use in last 30 days – NS  p=.151  Benzodiazepine use – NS  p=1.00  Cocaine use in last 30 days - NS  p=.714  Primary drug heroin– NS  p=.195  Alcohol use in last 30 days - NS p=.476 | Current methadone – NS  p=1.00Methadone dose – Ns  p=.696  Mean counselling session/week – S  p=.001  Attended any counselling – S  p=.005 | - | HIV+ - NS  p=.290  Week 1 positive opiate test (positive) – S  p=.002  Mean percentage opiate test – S  p=.002 | Marital Status – N/A  Employed (part or full time) less likely to drop out 30% vs 72.7% – S  p=.029  Lives with active user – NS  p= 1.00  Lives with children – NS  p=.125  Insurance type – private – NS  P= .378 | - |
| Strike 2005 (89) | Retention at 2 years (49.8%) | Retention at 2 years  Logistic regression  Odds Ratios (95% CI) | Age - S  1.09(1.05-1.13)  Age squared - S  0.999(0.98-0.99)  Age x group practice – S  0.98(0.98-0.99) | - | Group practice – S  4.19(2.76-6.35)  Group practice and correctional - S  5.57(4.25-7.30)  Group practice and individual practitioner - S  6.16(4.89-7.74)  Individual practitioner and correctional - S  3.09(2.41-3.95)  Group practice, individual practitioner and correctional - S  7.51(5.49-10.28)  Clinic x South region - S  2.43(1.07-5.55)  Clinic x East region - S  3.41(2.32-5.22)  Clinic x North region - S  0.39(0.17-0.89) | - | - | - | Central East – S  0.80(0.71-0.91)  Central South – S  0.79(0.67-0.93  Central West - S  1.27(1.09-1.47)  East - S  0.53(0.42-0.65)  North - S  1.76(1.31-2.36)  Episode - S  0.79(0.74-0.86)  Sex x group practice/independent – S  2.38(1.09-5.21)  Sex x group practice/correctional/independent – S  1.54(1.05-2.27) |
| Sullivan 2013 (90) | Dropout at 6 months (53%); at 12 months (66%) and 24 months (77%) | Retention (time on treatment – follow-up 2 years)  Accelerated failure time frailty models  Times Ratios (95% CI) | Gender (ref: female) – S  0.80(0.77-0.83)  Gender x dose (greater than or equal to 60mg) – S  0.92(0.86-0.98)  Ethnicity (ref: Han): non-Han – NS  1.03(0.99-1.08) | No of years using drugs – NS  1(1.0-1.0)  Drugs used (ref: heroin only) Other/mixed – S  1.35(1.24-1.46) | Median dose in final week (ref: <60 mg):  >= 60mg/day – S  2.12(1.96-2.30)  Age started MMT - S 1.02(1.01-1.02)  Mean attendance (ref: 2 days or less per week (p/w))  3 or 4 days p/w – S  0.67(0.58-0.76)  5 or more days p/w – S  0.26(0.24-0.29)  Dose x attendance  Dose >= 60mg/day x 3 or 4 days/week – NS  1.05(0.99-1.13)  Dose >= x >= 5 days/week – S  1.36(1.27-1.45)  Opiate test result prior to dropout or censoring (ref: positive) – S  2.15(2.03-2.27)  Dose x opiate test result – S  0.93(0.88-0.98)  Opiate test result x attendance (3 or 4 days p/w) – S  1.51(1.42-1.62)  Opiate test result x attendance (more than or equal to 5 days p/w) –  S  4.45(4.15-4.76)  Counsellor on staff – NS  1.05(0.99-1.10) | Injection drug use in 6 months prior to MMT (ref: no) – NS  0.98 (0.96-1.01) | HIV status (ref: negative) – NS  0.95(0.90-1.01)  Positive on ART- NS  1.15(0.98-1.36)  HCV status (ref: negative) – NS  0.99(0.97-1.02) | Marital status (ref: single) Married/cohabit – S  1.06(1.03-1.09)  Divorced – NS  0.99(0.95-1.03)  Widowed – NS  0.91(0.75-1.09)  Other - NS 1.06(.80-1.41)  Employment status (ref: unemployed) – NS  1.02(0.99-1.05)  Education (ref: illiterate)  Primary – NS  1.03(0.95-1.12)  Junior high - S  1.15(1.06-1.25)  Middle or high school – S  1.28(1.17-1.39)  Junior college or higher – S  1.40(1.25-1.56)  Dwelling (ref: family)  Friends – S  0.92(0.87-0.97)  Alone – S  0.91(0.88-0.94)  Other – NS  0.76(0.53-1.08)  Engagement in drug related crimes (ref: none reported)  Any – S  0.94(0.90-0.98) | Commute (less than or equal to 10 mins=0)  10-30 – NS  0.97(0.92-1.03)  31-60 – S  0.89(0.83-0.96)  >60 – S  0.83(0.75-0.91)  Commute x Attendance: Less than or equal to 10 mins x 3-4 days p/w – S  1.48(1.25-1.75)  10-30 mins x 3-4 d/w or less than 10 mins x 5 d/w – S  1.43(1.27-1.63)  31-60min x 3-4 d/w – S  1.36(1.18-1.58)  Greater than 60 min x 3-4 d/w S  1.27(1.16-1.40)  31-60 x 5 d/w – S  1.23(1.10-1.37) |
| Teoh 2017(91) | Retention at 1 year (94%) and 3 years (82%) | Dropout (follow-up 10 years)  Cox proportional hazards regression  Hazard Ratio (95% CI) | Age – NSU  Ethnicity – NSU | Other substance use in the month prior to MMT (ref: no): - NS  1.90 (0.97 – 3.73)  Quantity/frequency of heroin use in the month prior to MMT (OTI score) – NSU | Methadone dose (ref: < 40mg/day):  40 – 79mg/day – NS  0.79(0.33 – 1.89)  ≥ 80mg/day – S  0.23 (0.09 - 0.55)  Frequency of urine positivity within a year after initiation of MMT - NSU | Sexual partner – NSU  HIV risk-taking behaviour (OTI score) – S  1.06 (1.01 – 1.12) | HIV positive – NSU  Hepatitis C – NSU  Hepatitis B – NSU  Other medical illness – NSU  Health (OTI score) – NSU  Physical health (WHOQOL – BREF score) – NS  Psychological (WHOQOL – BREF score) – NS  0.97 ( 0.86 – 1.08)  Overall quality of life and general health (WHOQOL – BREF score) - NSU | Social functioning (OTI score)- NSU  Education level – NSU  Employment – NSU  Crime (OTI score) – NSU  Social relationships (WHOQOL – BREF score) – NSU  Environment (WHOQOL – BREF score) - NS  0.97 (0.88 – 1.08) |  |
| Wei 2013(92) | Cumulative retention rates at 1 year (87%); at 2 years (76%); at 3 years (66%); at 4 years (59%); at 5 years (49%); at 6 years (43%) | Dropout  Cox proportional hazard regression (adjusted for difference across clinics)  Odds Ratio (95% CI) | Female-S  0.75 (0.67-0.84)  Age – S  0.86 (0.80-0.91) | Drug use history of > 6 years- S  0.92 (0.88-0.97)  Positive urine tests (morphine)-S  0.34 (0.31-0.37) | Year of entry into treatment (ref 2006)  2007-S  1.58 (1.37-1.82)  2008-S  2.57 (2.22-2.99)  2009-S  3.36 (2.84-3.99)  2010-S  4.74 (3.84-5.84)  2011-S  5.46 (3.99-7.48)  Therapeutic dose (ref dose <30)  Dose >30- S  0.81 (0.76-0.87) | Needle sharing-S  0.86 (0.76-0.99) |  | Living status (ref with family)  With friends-S  1.45 (1.14-1.84)  Living alone-S  1.12 (1.00-1.26)  Being employed-S  0.93 (0.87-0.98) |  |
| Weinstein 2017 (93) | Retention for ≥ 1 year (45.7%) | Retained in treatment for ≥ 1 year  Generalized estimating equations (GEE) Logistic regression model  Odds ratio (95% CI) | Age at enrolment – S  1.19(1.05-1.34) per 10 year increase  Gender (ref: male) – S  1.55 (1.20-2.00)  Ethnicity (ref: white) – S Black - S  0.53 (0.36- 0.78)  Hispanic - S  0.66 (0.48-0.92)  Other – NS  2.03(0.57-7.17) | Benzodiazepine (illicit) use at enrolment – NSU  Cocaine use at enrolment – NS  0.86(0.61-1.22)  History of ever heroin use – NS  0.90(0.61-1.32)  Alcohol use at enrolment – NS  0.88(0.63-1.23) | Past buprenorphine treatment – NS  1.14(0.88-1.48)  Calendar year of treatment (ref:2003-2007):  2008-2010 – NS  0.91(0.70-1.18)  2011-2014 – S0.62(0.42-0.90)  Number OBOT period (ref: 1):  2^nd^ – S  0.39(0.28-0.53)  3^rd^ – S  0.34(0.18-0.64)  >4^th^ – NS  0.45(0.16-1.32) | - | Any Psychiatric diagnosis – S  1.75 (1.35- 2.27)  HCV antibody positive – S  0.59 (0.45-0.76) | Unemployment - S  0.72(0.56-0.92) | - |
| Yang 2013 (94) | Cumulative retention rate at 1 year (53%); 2 years (35%); 3 years (20%) | Dropout (Follow-up 5 years)  Cox proportional hazard models  Hazard Ratios (95% CI) | Age group (ref: ≤ 30) >30 – S  0.78(.69-.88)  Residence (ref: urban): rural – S  1.12(1.01-1.25) | Daily expense for drugs prior to MMT (ref:>300): ≤ 300 – S  0.80(0.71-0.90)  Drug use years prior to admission - NSU | Daily treatment dose (ref: >50):  ≤ 30 - S  1.44(1.29-1.61)  31-50 – S  1.33(1.21-1.48)  Re-enrolled (no=0): yes – S  1.41(1.34-1.49) | Sharing needles (ref: no): yes – S  1.23(1.08-1.40) | - | Income (ref: other): Family and friends – S  0.40(0.31-0.52)  Fixed income – S  0.40(0.30-0.54)  Temporary income – S  0.43(0.33-0.55)  Social welfare – S0.41(0.23-0.72)  Relationship with family (ref: bad): Good – S  0.68(0.58-0.80)  Communication with former drug taking peers last month(ref: yes):  No – S  0.90(0.84-0.98)  History of being arrested (ref: no): Yes – S  1.35(1.08-1.69)  Living with family - NSU | Considering treatment cost suitable (ref: no): yes – S  0.71(0.60-0.84)  Considering treatment operation time suitable (ref: no): yes - S  0.73(0.62-0.87) |
| Zhang 2015 (95) | Dropout  at 1 year (46.3%); 2 years (58.8%); 7 years (87.6%) | Dropout at 7 years  Cox Regression  Hazard Ratios (95% CI) | Age (10 years) – S  0.79 (0.72-0.87)  Gender – NSU | - | Low methadone dosage during the first treatment episode (<50 ml versus >50 ml – S  1.84 (1.64–2.06)  Higher proportion of positive urine test in first treatment episode (>50% versus<50%) – S  3.72 (3.30–4.20)  Last methadone dose - NSU | - | - | Married – NSU  Low education level (junior high or below versus otherwise) - S  1.21(1.05–1.40) | - |
| Zhou 2017(96) | Dropout  At 2 years (43.5% | Dropout at 2 years  Cox regression analysis to  Hazard ratios (95% CI) | Age – NR  Gender – NR | Drug using methods for past 6 months (ref Oral) - NR  Age at initial drug use (ref <= 20y) –NR  Initial morphine urine test result (ref Pos)  Repeated terminations (ref Yes)  Past treatment time (ref <1 year) | Detoxification prior to MMT – NR  Age at initial treatment (ref <=30y) | Sharing needles – NR | Clinical diagnosis:  SF-36v2MCS (HRQoL, mental health component – self reported, ref: ≤ 42) > 42 – NS  0.84(0.71-1.00)  SF-36v2PCS (HRQoL, physical health component – self reported, ref: ≤49)  > 49 – S0.83(0.69-0.98) | Marital Status – NR  Employment status – NR  Education – NR | QOL-DAv2.0 (QoL component – self reported) (ref: ≤66): > 66 – NS  0.86(0.72-1.03) |
| Zhou 2017(97) |  | Dropout (Follow-up to 7 years)  Cox PH regression  Hazard Ratios (95% CI) | Age (ref ≤ 30 years)- S  0.85(0.78-0.92)  41-50 yrs  0.80(0.73-0.88)  >50 yrs  0.74(0.60-0.91)  Gender (ref: male) – S  0.89(0.82-0.96) | Drug use type (ref: heroin) – S other  1.55(1.06-2.29)  Unauthorized drug use during MMT (ref: occasionally):  Intermittently – S  1.73(1.59-1.87)  Usually – S  3.89(3.60-4.20) | Average daily methadone dose (ref: >20 mg):  21-60mg – S  0.52(0.45-0.60)  >60 0.36(0.31-0.43)  Readmission (ref: yes) – S  1.18(1.12 -1.25) | Needle sharing experience (ref: yes) – S  0.89(0.81-0.99) | Initial morphine urine test (ref: positive) – S0.58(0.55-0.62) | Marital Status (ref: Married):  Other – S  1.08(1.02-1.14)  Employment status (ref: unemployed)– S  1.14(1.07-1.21)  Stable Income (ref: yes) – S  1.11(1.04-1.20)  Living with family (ref: yes) – S  1.11(1.04-1.19)  Contact with peer drug users over the past month (ref: yes) – S  0.72(0.67-0.77) | Time-taken to go to MMT clinic (ref: <30 min):  31-60 mins – S  1.16(1.07-1.25)  >60 mins – S  1.44(1.14-1.80)  Convenient MMT service time (ref: yes) – S  1.88(1.75-2.01)  Satisfaction with MMT service (ref: yes) – S  3.49(3.24-3.76) |
| Abbreviations:  NSU = Not significant in unadjusted analysis and hence not included in adjusted analysis  NS = Non statistically significant  S = Statistically significant  NR = Adjusted for in the model but results not reported in paper  Coefficients reported  CI = Confidence interval  Substance use  MMT = Methadone maintenance treatment  UDS+ = Urine drug screen positive  UDS- = Urine drug screen negative  Treatment Factors  Mg = Milligrams  OBOT = Office based opioid treatment  THD = Take home dose  Health Risk Behaviour  IDU = Injecting drug use  Health Symptoms  SF-36vMCS = Short form health survey: mental component summary  HRQoL = Health related quality of life  SF36vPCS = Short form health survey: physical component summary  HCV = Hepatitis C virus  HIV = Human immunodeficiency virus  TB = Tuberculosis  ART = Antiretroviral therapy  Social Functioning  APGAR = Adaptation, Partnership, Growth, Affection, Resolve  TANF = Temporary aid to needy families  GAX = General assistance presumptive disability  GAU = Financial assistance for low-income people without children  Other variables investigated in included studies  QOL – Dav2.0 = Quality of Life Scale for Drug Addicts  Mins = Minutes  D/W = days per week  RCQ-12 = Readiness to change questionnaire | | | | | | | | | |
